# Genome-wide detection of signatures of selection in indicine and Brazilian locally adapted taurine cattle breeds using whole-genome re-sequencing data

**DOI:** 10.1186/s12864-020-07035-6

**Published:** 2020-09-11

**Authors:** Elisa Peripolli, Christian Reimer, Ngoc-Thuy Ha, Johannes Geibel, Marco Antonio Machado, João Cláudio do Carmo Panetto, Andréa Alves do Egito, Fernando Baldi, Henner Simianer, Marcos Vinícius Gualberto Barbosa da Silva

**Affiliations:** 1 São Paulo State University (Unesp), School of Agricultural and Veterinarian Sciences, Jaboticabal, 14884-900 Brazil; 2 Animal Breeding and Genetics Group, Department of Animal Sciences, University of Goettingen, Albrecht-Thaer-Weg 3, 37075 Goettingen, Germany; 3 Center for Integrated Breeding Research, University of Goettingen, Albrecht-Thaer-Weg 3, 37075 Goettingen, Germany; 4 National Council for Scientific and Technological Development (CNPq), Lago Sul, 71605-001 Brazil; 5 Embrapa Dairy Cattle, Juiz de Fora, 36038-330 Brazil; 6 Embrapa Beef Cattle, Campo Grande, 79106-550 Brazil

**Keywords:** *Bos taurus indicus*, *Bos taurus taurus*, Signatures of selection, Local adaptation, Next-generation sequencing

## Abstract

**Background:**

The cattle introduced by European conquerors during the Brazilian colonization period were exposed to a process of natural selection in different types of biomes throughout the country, leading to the development of locally adapted cattle breeds. In this study, whole-genome re-sequencing data from indicine and Brazilian locally adapted taurine cattle breeds were used to detect genomic regions under selective pressure. Within-population and cross-population statistics were combined separately in a single score using the de-correlated composite of multiple signals (DCMS) method. Putative sweep regions were revealed by assessing the top 1% of the empirical distribution generated by the DCMS statistics.

**Results:**

A total of 33,328,447 biallelic SNPs with an average read depth of 12.4X passed the hard filtering process and were used to access putative sweep regions. Admixture has occurred in some locally adapted taurine populations due to the introgression of exotic breeds. The genomic inbreeding coefficient based on runs of homozygosity (ROH) concurred with the populations’ historical background. Signatures of selection retrieved from the DCMS statistics provided a comprehensive set of putative candidate genes and revealed QTLs disclosing cattle production traits and adaptation to the challenging environments. Additionally, several candidate regions overlapped with previous regions under selection described in the literature for other cattle breeds.

**Conclusion:**

The current study reported putative sweep regions that can provide important insights to better understand the selective forces shaping the genome of the indicine and Brazilian locally adapted taurine cattle breeds. Such regions likely harbor traces of natural selection pressures by which these populations have been exposed and may elucidate footprints for adaptation to the challenging climatic conditions.

## Background

The first cattle herds were brought to Brazil by Portuguese conquerors in 1534 during the Brazilian colonization period [1]. These cattle have undergone to a process of natural selection for more than 450 years in a wide range of ecosystems throughout the country [2]. Natural selection in a remarkably diverse set of environments together with recurring events of breed admixture led to the development of locally adapted cattle breeds, i.e. Curraleiro Pé-Duro, Pantaneiro, Crioulo Lageano, Caracu, and Mocho Nacional [3]. By the end of the nineteenth century, the increasing demand for food supply triggered the imports of exotic and more productive breeds of indicine origin [3, 4]. As a consequence, a reduction in locally adapted cattle breed populations has occurred to such an extent that nowadays, most of them are threatened with extinction [3, 5].

Brazilian locally adapted cattle breeds have been subjected to strong environmental pressures and faced several difficulties including hot, dry or humid tropical climate conditions, scarce food availability, diseases, and parasite infestations without any significant selective pressure imposed by man [2]. Influenced by the environment and shaped by natural selection, these animals acquired very particular traits to thrive in distinct ecosystems, which has presumably left detectable signatures of selection within their genomes. In this regard, Brazilian locally adapted cattle breeds represent an important genetic resource for the understanding of the role of natural selection in diverse environments, providing new insights into the genetic mechanisms inherent to adaptation and survivorship [6]. Although their productivity is much lower compared to highly-specialized breeds under intensive production systems [7, 8], great efforts have been made to improve our knowledge of locally adapted breeds [5, 9, 10] and their use in crossbred schemes.

According to Utsunomiya et al. [11], signatures of selection studies should strongly focus on small local breeds given their endangered status and the putative importance of their genomes in unraveling footprints of selection by elucidating genes and structural variants underlying phenotypic variation. Advances in molecular genetics and statistical methodologies together with the availability of whole-genome re-sequencing has notably improved the accuracy to disentangle the effects of natural and artificial selection in the genome of livestock [12–14]. However, despite the recent achievements in high-throughput sequencing, studies to detect positive selection in endangered Brazilian locally adapted cattle breeds are incipient. Previous studies on such breeds have mainly focused on population structure and genetic diversity using Random Amplified Polymorphic DNA (RAPD), pedigree data, microsatellite, and Single-Nucleotide Polymorphism (SNP) arrays [15–19].

In this study, we report for the first-time signatures of selection derived from whole-genome re-sequencing data in three Brazilian locally adapted taurine cattle breeds as well as in one indicine breed. Potential biological functions of the genes screened within the putative candidate regions were also examined to better elucidate the phenotypic variation related to adaptation shaped by natural selection.

## Results

### Data

DNA samples from 13 Gir (GIR), 12 Caracu Caldeano (CAR), 12 Crioulo Lageano (CRL), and 12 Pantaneiro (PAN) re-sequenced to 15X genome coverage were used. An average alignment rate of 99.59% was obtained. After SNP calling and filtering, a total of 33,328,447 SNPs distributed across all 29 autosomes were retained for subsequent analyses with an average read depth of 12.37X (9.57 ~ 17.52X).

### Variant annotation and enrichment

Of the total SNPs identified (*n = 33,328,447 SNPs*), most of them were located in intergenic (67.17%) and intronic (25.85%) regions (Additional file 1). A total of 1,065,515 (3.19%) variants were located in the 5-kb regions upstream from genes, and 928,061 (2.78%) in the 5-kb regions downstream from genes. Several variants with high consequence on protein sequence were identified, including splice acceptor variant (*n = 471*), splice donor variant (*n = 481*), stop gained (*n = 1111*, stop lost (*n = 58*), and start lost (*n = 208*). According to SIFT scores, 24,159 variants (23,428 missense, 578 splice region, and 143 start lost) were classified as deleterious.

Following variant annotation, we further investigated the gene content within the predicted variants to cause relevant biological functions. A total of 1189 genes were described within variants with high consequence on protein sequence and 7373 genes within those causing a deleterious mutation based on the SIFT score. Functional enrichment analysis revealed several gene ontology (GO) terms and one Kyoto Encyclopedia of Genes and Genomes (KEGG) pathway overrepresented (*p* < 0.01) for the set of genes previously described (Additional files 2 and 3), however, none of them have been associated with the traits/phenotypes that could be affected by the natural selection which those breeds have been subjected to.

### Population structure

**Fig. 1:**
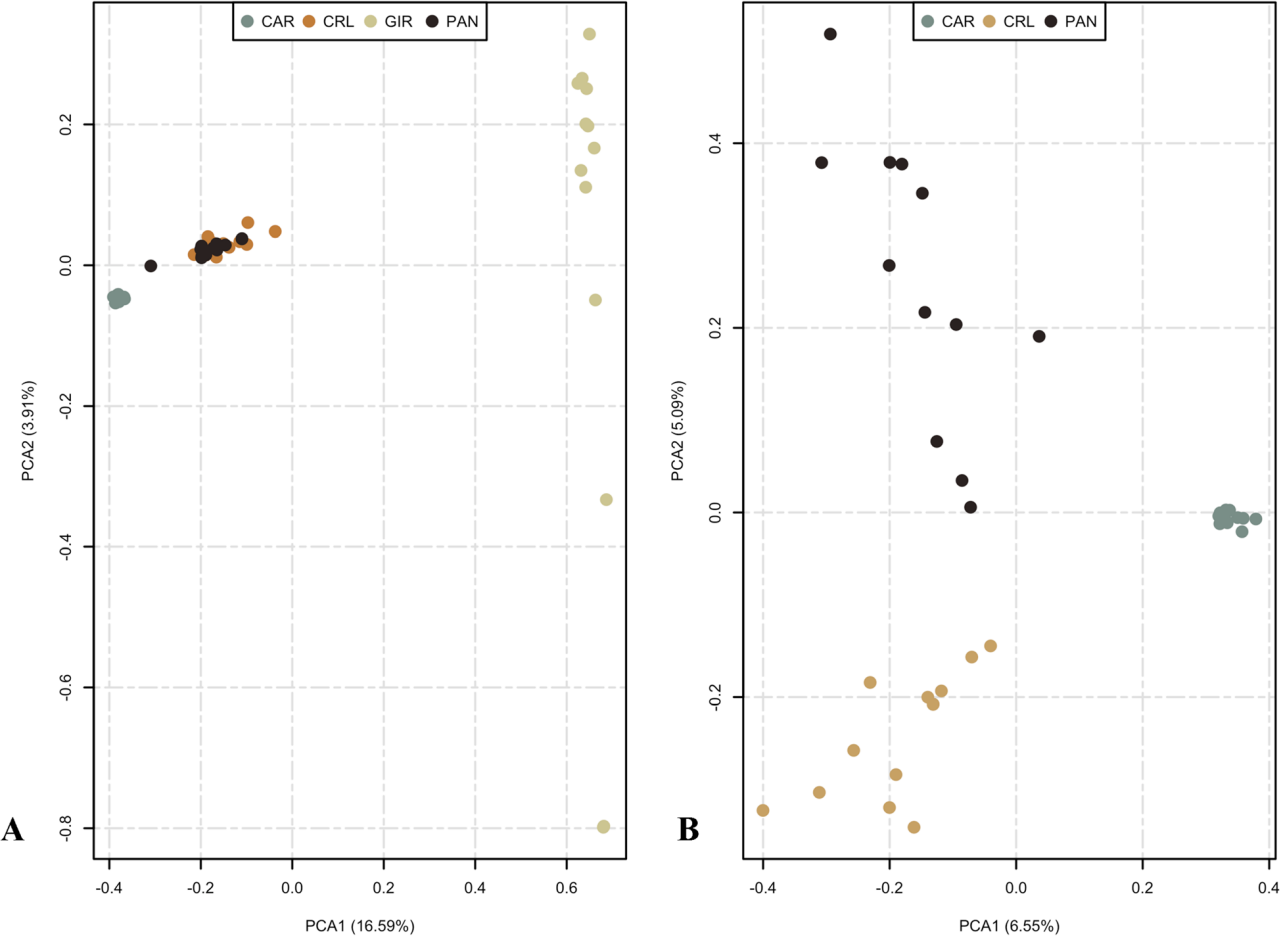
Principal components analysis (PCA) scores plot with variance explained by the first two principal components in brackets. **a** PCA scores for the four breeds (Caracu Caldeano – CAR, Crioulo Lageano – CRL, Gir – GIR, and Pantaneiro - PAN. **b** PCA scores for the locally adapted taurine cattle breeds (Caracu Caldeano – CAR, Crioulo Lageano – CRL, and Pantaneiro – PAN)

The population structure among breeds was dissected by analyzing the first two principal components, which accounted for roughly 20% of the genetic variability and divided the populations into three clusters (Fig. 1a). A clear separation could be observed between indicine (*Bos taurus indicus*) and locally adapted taurine (*Bos taurus taurus*) populations. Within the taurine populations, the greatest overlap of genetic variation was observed between CRL and PAN breeds. Despite clustering together, the analysis of molecular variance (AMOVA) revealed genetic differentiation between those two breeds (*p* < 0.001, Additional file 4), indicating that all four breeds could be considered as genetically independent entities. Further, when analyzing the first two principal components encompassing the locally adapted taurine cattle breeds (Fig. 1b), an evident separation could be observed between CAR and the remaining two populations. The analysis also distinguished CRL from PAN, agreeing with the AMOVA results.

**Fig. 2:**
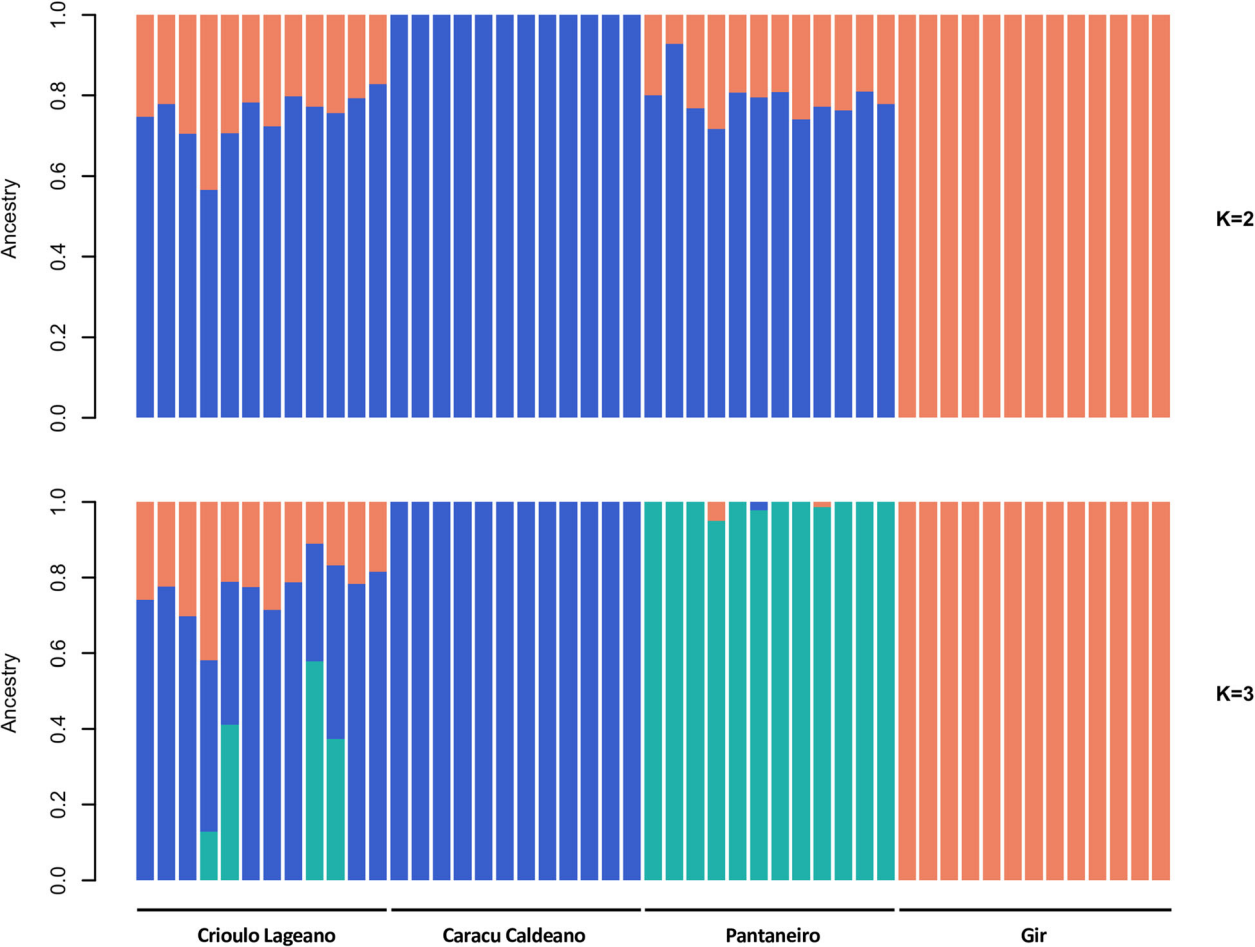
Population structure inferred by using the ADMIXTURE software. Each sample is denoted by a single vertical bar partitioned into *K* colors according to its proportion of ancestry in each of the clusters. Ancestral contributions for *K = 2* and *K = 3* are graphically represented

Admixture analysis was performed to further estimate the proportions of ancestry (K) in each population (Fig. 2). The lowest cross-validation error (0.387) was observed for *K = 2*, revealing the presence of two main clusters differentiating the locally adapted taurine populations from the indicine population. Within the taurine populations, the CAR breed did not show admixed ancestry while CRL and PAN breeds showed 77% of taurine and 23% of indicine ancestry on average. When *K = 3* was assumed, CRL samples revealed evidence of admixed ancestry from other breeds, whereas PAN samples were quite homogeneous, with little indication of introgression from other breeds. CAR and GIR breeds displayed a greater uniformity and did not reveal major signs of admixture of other breeds, being consistent with *K = 2*.

### Genomic inbreeding

Descriptive statistics for runs of homozygosity-based inbreeding coefficients (F_ROH_) are shown in Table 1. The average inbreeding coefficients did not differ significantly (*p* < 0.05) among breeds, with the exception of CAR animals. It is worth to highlight that these animals also displayed the smallest inbreeding variability among all breeds, supported by the lowest coefficient of variation.

**Table 1:** Descriptive statistics of runs of homozygosity-based inbreeding coefficient (F_ROH_) for Gir (GIR), Crioulo Lageano (CRL), Caracu Caldeano (CAR), and Pantaneiro (PAN) cattle breeds

Breed	Mean	Median	Minimum	Maximum	Coefficient of variation (%)
Gir	0.040^b^	0.038	0.020	0.060	29.37
Crioulo Lageano	0.036^b^	0.028	0.017	0.082	53.69
Caracu Caldeano	0.138^a^	0.140	0.121	0.153	8.63
Pantaneiro	0.045^b^	0.042	0.022	0.096	43.56

Means sharing a common letter within a column were not significantly different (p < 0.05) from one another

### Selective sweeps

**Fig. 3:**
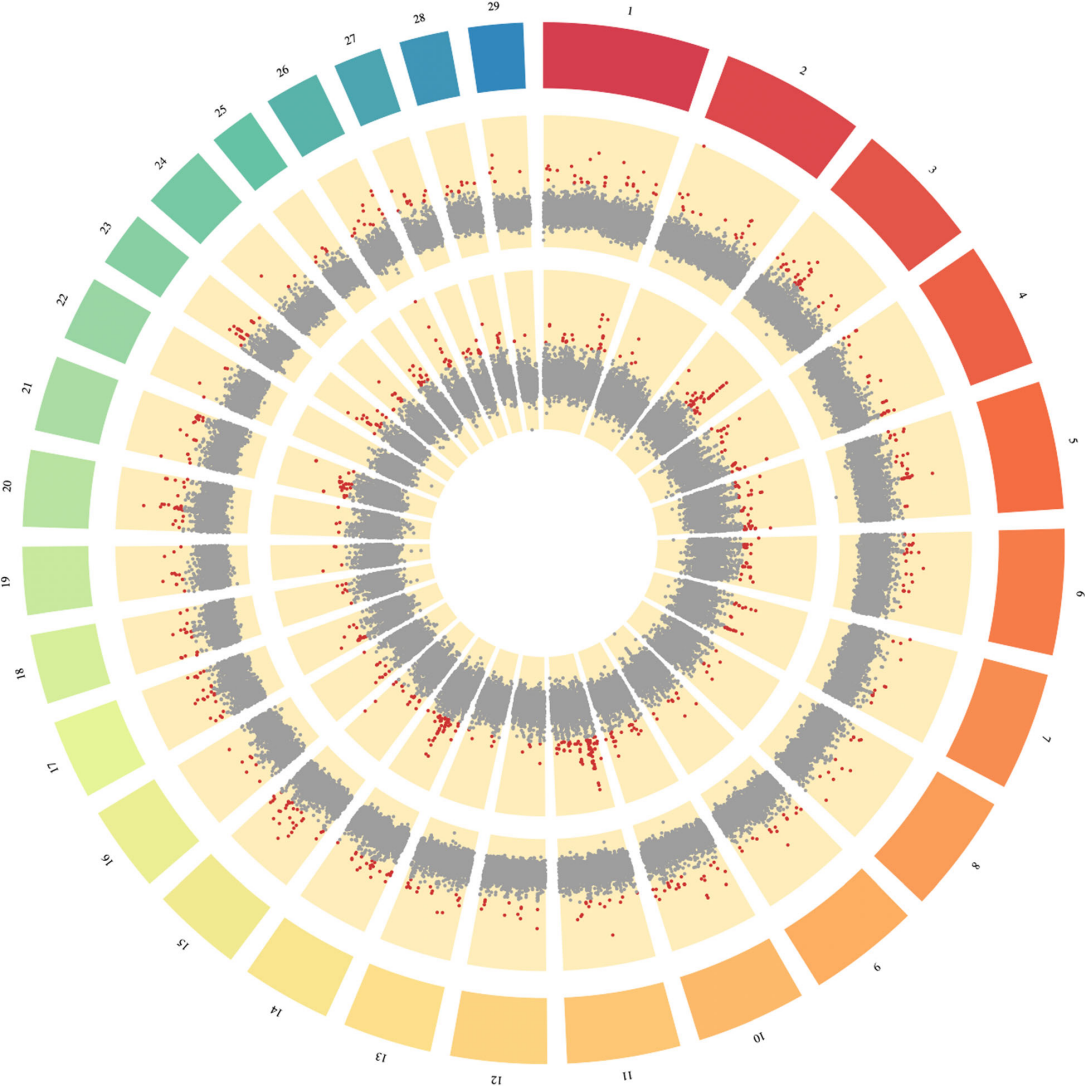
Whole-genome signatures of selection for the within-population DCMS statistic (outer circle) and cross-population DCMS statistic (inner circle). The x-axis shows the window position along the chromosome, and the y-axis the DCMS value associated with such window. Reds dots correspond to the top 1% of the empirical distribution generated by the DCMS statistics

A total of 499 putative sweep regions encompassing 221 genes were identified from the top 1% of the empirical distribution generated by the within-population de-correlated composite of multiple signals (DCMS) statistic [20] (Fig. 3, Additional file 5). For the cross-population DCMS statistic, the top 1% of the empirical distribution revealed 503 putative sweep regions comprehending 242 genes (Additional file 6). The *Bos taurus* autosome (BTA) 3 displayed the highest number of putative sweep regions for the within-population DCMS statistic (*n = 33*), while BTA11 did for the cross-population DCMS statistic (*n = 67*). The functional importance of the annotated genes was assessed by performing GO and KEGG pathway enrichment analysis separately for each DCMS statistic and its respective retrieved gene list. No overall significant enrichment of any particular GO nor KEGG was found after adjusting the *p*-values for False Discovery Rate [21].

Five genomic regions overlapped between the candidate sweep regions of the within-population and cross-population DCMS statistics (BTA4:101600000–101,650,000, BTA5:3700000–3,750,000, BTA9:98650000–98,700,000, BTA11:22300000–22,350,000, and BTA11:53900000–53,950,000). When inspecting in detail, the region on BTA4:101600000–101,650,000 harbored two quantitative trait locus (QTL) with functions related to the bovine respiratory disease [22] and body condition score [23]. The remaining four regions have not been associated with any QTL in cattle so far, however, they were found to be in close vicinity (~ 15 to 237 kb) with specific QTLs for beef cattle production traits. Such QTLs included body weight at yearling, calving ease, body weight gain, and marbling score [24–26]. Further, among the five overlapping candidates sweep regions, only the one on BTA9 was found to harbor a gene, the *PRKN*.

### Selective sweeps and runs of homozygosity

Shared genomic regions harboring several protein-coding genes were identified between runs of homozygosity (ROH) hotspots and the putative sweep regions retrieved from the DCMS statistics (Table 2). ROH hotspots for each breed are described in Additional file 7. For the shared regions disclosed when considering the within-population DCMS statistic, the ones located on BTA1:8300000–8,350,000 and BTA1:41600000–41,650,000 coincided with a QTL for somatic cell score [27] and maturity rate [28], respectively. It is noteworthy to underscore that despite not displaying any overlapping QTL, the region on BTA8:15700224–15,700,228 was described nearby (~ 99 kb) a QTL for tick resistance [29], and those on BTA21:6550000–6,600,000 and BTA21:63250000–63,300,000 were very close (< 14 kb) to QTLs for reproductive-related traits [30, 31]. When considering the cross-population DCMS statistic, the candidate regions overlapped previously identified QTLs formerly implicated in dairy-related [35–37, 39] and body-related (weight [24], energy content [34], and conformation [35]) traits. Further, several QTLs associated with body conformation and growth [23, 24, 33], reproductive-related traits [28, 32], and coat texture [38] were described to be in very close proximity (~ 18.98 to 88.38 kb).

**Table 2:** Gene annotation and reported QTLs for the shared genomic regions between runs of homozygosity (ROH) hotspots and the putative sweep regions retrieved from the within-population and cross-populations DCMS statistics

BTA^**1**^	Start	End	Genes	QTL^2^
*Within-population DCMS statistic x ROH*				
1	8,300,000	8,350,000	–	**Somatic cell score** [27]
1	41,600,000	41,650,000	*EPHA6*, *ARL6*	**Maturity rate** [28]
1	112,250,000	112,300,000	*KCNAB1*	–
8	15,800,000	15,850,000	*–*	Tick resistance [29]
15	35,365,655	35,399,999	*OTOG*	–
15	35,400,001	35,450,000	–	–
18	34,718,675	34,750,000	*CDH16*, *RRAD*	–
21	6,550,000	6,600,000	*ADAMTS17*	Calving ease [30]
21	63,250,000	63,300,000	*VRK1*	Interval to first estrus after calving [31]
*Cross-population DCMS statistic x ROH*				
3	77,250,000	77,300,000	–	Body condition score [23]
5	31,800,000	31,850,000	–	Body weight (yearling) [24], Conception rate [32]
5	38,761,637	38,761,745	*YAF2*	
7	57,050,000	57,100,000	–	Rump angle [33]
11	67,450,000	67,500,000	*ANTXR1*, *GFPT1*	**Body weight (yearling)** [24]**, Body energy content** [34]
11	67,700,000	67,749,999	–	–
11	67,750,001	67,800,000	*NFU1*	–
11	68,550,000	68,600,000	*PCYOX1*	–
14	52,900,000	52,914,848	–	Maturity rate [28]
15	10,150,000	10,200,000	–	–
15	10,900,000	10,950,000	–	**Calving ease (maternal)** [35]**, Daughter pregnancy rate** [35]**, Foot angle** [35]**, Milk fat percentage** [35]**, Milk fat yield** [35]**, Net merit** [35]**, Length of productive life** [35]**, Milk protein percentage** [35]**, Milk protein yield** [35]**, Calving ease** [35]**, Somatic cell score** [35]
20	38,000,000	38,050,000	*RANBP3L*, *NADK2*	**Milk protein percentage** [36]**, Milk protein yield** [37]**, Milk yield** [37]**,** Coat texture [38]
21	200,000	250,000	–	–
25	1,345,564	1,350,000	*NME3*, *MRPS34*	**Milk fat yield** [39]

^1^ BTA: *Bos taurus* autosome; ^2^ QTLs within the candidate genomic regions are highlighted in bold. Non-bold QTLs were the closest and most suitable candidate QTL for the given candidate region

### Overlap with candidate regions under positive selection in other cattle populations

Several putative sweep regions identified from the top 1% of the empirical distribution generated by the within-population and cross-population DCMS statistics were in agreement with previous research on signatures of selection in cattle (Additional files 8 and 9, respectively). Such studies included indigenous African and Spanish [6, 40–43], native [44–46], tropical-adapted [6, 47–49], Chinese [49, 50], and commercial beef and dairy [13, 41, 49, 51–54] cattle breeds. For the five genomic regions identified overlapping in between the DCMS statistics, the one on BTA9:98650000–98,700,000 matched with a previous study on cattle breeds selected for dairy production [54]. Besides, common signals found between ROH hotspots and the within-population and cross-population DCMS statistics were also supported by previously published data on signatures of selection [6, 41, 43, 44, 46, 50, 53] (Additional files 10 and 11, respectively).

## Discussion

### Population structure

The segregation between indicine and taurine cattle populations described in both principal component and admixture analysis (*K = 2*) reflects the divergence and evolutionary process started roughly two million years ago [55, 56]. As a result of the domestication process and selective breeding over time, the cattle can be classified into temperate (*Bos taurus taurus* or taurine) and tropical (*Bos taurus indicus* or indicine) based on the common adaptive and evolutionary traits they have acquired [57]. Within the Brazilian locally adapted taurine breeds, the principal component analysis (PCA) indicates the highest relatedness between CRL and PAN breeds and their divergence from the CAR breed may be explained by the European cattle type introduced in Brazil during the colonization period [58]. These results were similar to those obtained using RAPD [17] and microsatellites [19]. Portuguese purebred cattle brought to Brazil belonged to three different bloodlines: *Bos taurus aquitanicus*, *Bos taurus batavicus*, and *Bos taurus ibericus*. In this regard, CRL and PAN breeds descended from a common ancestral pool and have their origin in breeds from *Bos taurus ibericus* cattle, while the CAR cattle is derived from the *Bos taurus aquitanicus* cattle [17]. Further, the divergence within the locally adapted cattle breeds may be a result of artificial selection events over time since the CAR cattle have been selected for milk production for the past 100 years, while CRL and PAN started recently to be artificially selected.

Levels of introgression of indicine genes in taurine breeds described herein are consistent with previous studies on Brazilian locally adapted taurine breeds [16, 17, 19]. This gene flow reinforces the concept that the import of exotic breeds at the beginning of the twentieth century [3] led to the miscegenation of the locally adapted breeds due to crossbreeding practices, resulting nearly in their extinction [4]. In this regard, the CRL breed experienced some introduction of Nellore (*Bos taurus indicus*) genes for a short period in the eighties [17], which can be visualized when assuming *K = 2* and *K = 3*. Concurring with our findings, Egito et al. [19] also revealed that CRL and PAN animals were the closest to the indicine cattle among four Brazilian locally adapted cattle breeds, displaying the highest frequency of indicine gene introgression. A cytogenetic analysis study on the PAN cattle also revealed absorbing crosses with the indicine cattle [59]. In addition, the absence of admixture patterns in CAR individuals has been previously described by Campos et al. [16] and Egito et al. [21]. The homogeneity of such population most likely reflects its formation process and the objective of selection for dairy traits since 1893 [60], which may have distinguished them from other locally adapted taurine breeds when taking into consideration the genetic structure integrity.

### Genomic inbreeding

As already stated, the Brazilian locally adapted cattle breeds nearly disappeared between the late 19th and beginning of the twentieth century, and most of them are nowadays threatened with extinction [3, 5]. It is worth to stress out that the CAR cattle are an exception, and they can be considered as an established breed [5, 61]. In this regard, animals comprising our dual purpose cattle populations, which were exploited for meat production in former times [62], are nowadays mainly used in animal genetic resources conservation programs (in situ and ex situ) and as a germplasm reservoir to preserve the genetic variability [4, 63]. Different from the dual-purpose cattle populations, the dairy populations are no longer considered endangered, and such animals have been selected for milk production traits in the southeastern region of Brazil since 1893 (CAR, [60]) and the early nineties (GIR, [64]).

Most of the locally adapted cattle breeds in Brazil developed from a narrow genetic base, and in such cases, inbreeding can increase over generations and reduce genetic variability [65]. Despite their population background, CRL and PAN animals displayed low F_ROH_ estimates, concurring with heterozygosity estimates (Results not shown). Decreased levels of inbreeding and high genetic variability have been previously described for both breeds, probably resulting from a slight selection pressure and herd management focused on maintaining genetic diversity by using a male:female relationship larger than usual [19]. Egito et al. [15] attributed such results to the formation of new PAN herds from 2009 onwards while Pezzini et al. [18] associated it with the diversification in the use of CRL sires. Further, Egito et al. [19] stated that CRL and PAN cattle were the most diverse population with the highest mean allelic richness among four locally adapted cattle breeds investigated. Such results are consistent with F_ROH_ estimates found in this current work, reflecting mild selection pressure in our dual-purpose cattle populations together with rationale mating decisions and herd management taken by the breeders and associations.

The highest F_ROH_ found for the CAR population most likely reflects its history of selective breeding for milk-related traits from a limited genetic base and the occurrence of a population decrease in the sixties, as discussed by Egito et al. [19]. According to Marras et al. [66], it is not unusual to disclose a higher sum of ROH in dairy than in beef populations. In this regard, the reduction of genetic variability through the increase of autozygosity in dairy breeds can be explained by the intense artificial selection with the use of a relatively small number of proven sires [67]. Despite being also specialized for milk-related traits, it is not surprising that the GIR population did not show as high F_ROH_ levels as did CAR. Previous studies have also shown low inbreeding rates for the GIR cattle considering pedigree-based inbreeding coefficient [68, 69] and F_ROH_ [70, 71]. A trend in the decrease of inbreeding has been previously described [68, 70], and it happens along with the establishment of the Brazilian Dairy Gir Breeding Program (PNMGL) and the Gir progeny testing. Presumptively, these two concomitant events led to the dissemination of the breed, allowing formerly closed herds to start using semen of proven sires, increasing the overall genetic exchange and reducing the average inbreeding over time.

### Candidate regions under positive selection

After combining the top 1% putative sweep regions retrieved from the within-population and cross-population DCMS statistics, five candidate regions harboring two QTLs and only one protein-coding gene were identified. Such results allowed us to highlight the body condition score QTL [23] on BTA4:101600000–101,650,000, which can be defined as the amount of metabolized energy stored in fat and muscle of a live animal [72]. During periods of energy shortage, key hormones expression and tissue responsiveness adjust to increase lipolysis to meet energy requirements and maintain physiological equilibrium [73, 74]. Regulation and coordination of energy partitioning and homeostasis is a challenge to sustainable intensification of cattle productivity in the tropics. The variation in the animal’s nutritional and energetic balance may explain the observed variability in performance between animals in different environments [75]. Negative energy balance most likely reduce energy expenditure, impairing reproductive performance [76], and increasing the susceptibility to infections [77]. As formerly described, the Brazilian locally adapted cattle breeds faced several environmental pressures to thrive in the tropics under harsh environmental conditions, suggesting that animals that were able to minimize the mobilization of adipose tissue reserves in response to the energy deficit might have conferred fitness advantage than the average individual in the given population.

The *PRKN* (also known as *PARK2*) was the only annotated gene identified in between the DCMS statistics, and its functions have been associated with adipose metabolism and adipogenesis [78]. Remarkably, it is considered a strong positional candidate for adiposity regulation in chicken [79].

We also explored common signals between ROH hotspots and the top 1% putative sweep regions retrieved from both DCMS statistics to increase the power of signals. Among the genes identified when considering the within-population DCMS statistic, we revealed the presence of two interesting genes that have been described to have effects on temperament (*EPHA6*) [80] and body size (*ADAMTS17*) [81] in cattle. Further, one gene associated with temperament (*ANTXR1*) [82] was also highlighted when considering the cross-population DCMS statistic.

In tropical and subtropical regions, cattle productivity depends not only on the inherent ability of animals to grow and reproduce but also on their ability to overcome environmental stressors that impact several aspects of cattle production [83]. In cattle, stress responsiveness has been associated with cattle behavior, more specifically, temperament. Temperament can adversely affect key physiological processes involved in cattle growth, reproduction, and immune functions [84]. Studies have shown that non-temperamental cattle tend to gain weight faster [85–87], spend more time eating [87], and have a higher dry matter intake and average daily gain [85, 88] than temperamental cattle. Further, studies have discussed the negative impacts of temperamental animals on immune-related functions (reviewed by [84]). Two reasons might explain those genes associated with temperament located on ROH hotspots overlapping regions on BTA1:41600000–41,650,000 and BTA11:67450000–67,500,000. The first reason is that such genes likely reflect levels of introgression of indicine genes in locally adapted taurine cattle breeds, as confirmed by admixture analysis. *Bos taurus indicus* and their crosses have been reported to be more temperamental than *Bos taurus taurus* cattle when reared under similar conditions [89]. The second reason is that the locally adapted taurine cattle breeds were able to overcome environmental stressors through natural selection over time and could prosper in such harsh tropical environment.

The *ADAMTS17* gene, described enclosing a ROH hotspot overlapping region on BTA21:6550000–6,600,000, is a well-known candidate gene with a major impact on body size [81, 90, 91]. Much has been discussed about the relationship between body size and environmental adaptation. Variations in body size may be explained as an adaptive response to climate and/or can be driven by changes in feed resources and seasonal influences [92, 93]. In this regard, large body size animals can better tolerate austere conditions, having advantages under cold stress as well as in the use of abundant forage resources [94]. On the other hand, smaller animals exhibit better adaptation to warmer and dry climates [95–97] and are more efficient for grazing under seasonal and scarce forage resources [98]. Based on morphological measurements, it should be noted that the indicine and Brazilian locally adapted taurine cattle breeds are small to medium-sized breeds. Both GIR, CRL, and PAN have reduced body size and lightweight, in which females exhibit an average adult live weight of 418 kg [99], 430 kg [100], and 298 kg [101], respectively. CAR animals have a greater body size among the locally adapted cattle breeds, with females displaying an average live weight of 650 kg [102].

Two intersecting QTLs associated with productivity traits usually favored in commercial breeds (somatic cell score and maturity rate QTLs) were found in ROH hotspots overlapping regions when considering the within-population DCMS statistic. Among the QTLs identified when considering the cross-population DCMS statistic, the one associated with body energy content [34] must be highlighted given its importance in energy partitioning and homeostasis, as previously discussed. Additionally, several remarkably QTLs neighboring the candidate regions intervals were identified. These QTLs have been associated with different biological functions linked to local environment adaptation, such as parasite vector resistance (tick resistance QTL), reproductive-related traits (calving ease, interval to first estrus after calving, conception and maturity rate QTLs), body conformation and morphology traits (body condition score, body weight at yearling, rump angle QTLs), and coat color (coat texture QTL).

The genes and QTLs identified within the candidate regions provide a hint about the selective forces shaping the genome of the indicine and Brazilian locally adapted taurine cattle breeds. Such selective forces were described to be likely associated with adaptation to a challenging environment and environmental stressors. Further, several QTLs identified nearby the candidate regions intervals were also associated to a lesser extent with beef cattle production traits, while others with various biological functions presumably linked to selection to environmental resilience as well.

### Overlap with candidate regions under positive selection in other cattle populations

The greatest number of the putative sweep regions identified from the top 1% of the within-population DCMS statistic overlapped with candidate regions under positive selection previously reported in five cattle breeds selected for dairy production [54], comprehending roughly 22% (*n = 52*) of the overlapping regions. For the top 1% of the cross-population DCMS statistic, the greatest number was described for native cattle breeds from Siberia, eastern and northern Europe [46], totaling nearly 17% (*n = 50*) of the overlapping regions. Remarkably, in both statistics, the majority of the shared signals within those reported in the literature was found associated with specialized cattle breeds (i.e. dairy and beef). We also identified signatures of selection within those reported in the literature shared by breeds showing different production selection within the same candidate region. According to Gutiérrez-Gil et al. [103], such genomic regions may reflect selection for general traits such as metabolic homeostasis, or they might disclose the pleiotropic effects of genes on relevant traits underlying specialized cattle breeds.

The greater number (seven out of 11) of the putative sweep regions shared between ROH hotspots and the top 1% putative sweep regions retrieved from both DCMS statistics overlapped with regions previously described on local and native cattle breeds [41, 43, 44, 46]. Such results allow us to assume that the same selective forces are most likely acting across these populations, and such regions might have been shaped by selection events rather than genetic drift or admixture events.

It is noteworthy to underscore that the regions under positive selection for other cattle populations reported herein were mainly obtained through medium and high-density SNP arrays. SNP genotyping arrays suffer from SNP ascertainment bias, and it strongly influences population genetic inferences (reviewed by Lachance and Tishkoff [104]). Besides, some scan methodologies based on site frequency spectrum and population differentiation may be more likely to ascertainment bias than others [105, 106], compromising the power of the tests and may yielding to flawed results [107] when compared to those obtained from whole-genome re-sequencing data.

## Conclusions

By using whole-genome re-sequencing data, we identified candidate sweep regions in indicine and Brazilian locally adapted taurine cattle breeds, of which the latter have been exposed to a process of natural selection for several generations in extremely variable environments. The signatures of selection across the genome could provide important insights for the understanding of the adaptive process and the differences in the breeding history underlying such breeds. Our findings suggest that admixture has occurred in some locally adapted taurine populations due to the introgression of exotic breeds, and the stratification results revealed the genetic structure integrity of the dairy populations sampled in this study. Candidate sweep regions, most of which overlapped with or were nearby reported QTLs and candidate genes closely linked to cattle production traits and environmental adaptation. Putative sweep regions together with ROH hotspots also provided valuable shreds of evidence of footprints for adaptation to the challenging climatic conditions faced by the breeds. The candidate sweeps regions and the gene list retrieved from them can improve our understanding of the biological mechanisms underlying important phenotypic variation related to adaptation to hostile environments and selective pressures events to which these breeds have undergone. Furthermore, the study provides complementary information which could be used in the implementation of breeding programs for the conservation of such breeds.

## Methods

### Samples, sequencing, and raw data preparation

Sequencing analysis was based on data from 13 Gir (*Bos taurus indicus*, dairy production use), 12 Caracu Caldeano (*Bos taurus taurus*, dairy production use), 12 Crioulo Lageano (*Bos taurus taurus*, dual purpose use), and 12 Pantaneiro (*Bos taurus taurus*, dual purpose use) animals. The studied breeds can be classified into two groups: (i) indicine breeds represented by the Gir (GIR) cattle; and (ii) locally adapted taurine cattle breeds encompassing Caracu Caldeano (CAR), Crioulo Lageano (CRL), and Pantaneiro (PAN) cattle. Animals were sampled from three Brazilian geographical regions, including the south (CRL), southeast (GIR and CAR), and mid-west (PAN) (Additional file 12).

DNA was extracted from semen samples that were collected from GIR bulls and blood samples from the remaining breeds. The semen straws were acquired from three commercial artificial insemination centers (American Breeders Service (ABS), Cooperatie Rundvee Verbetering (CRV), and Alta Genetics) and the DNA samples from the Animal Genetics Laboratory (AGL) at EMBRAPA Genetic Resources and Biotechnology (Cenargen, Brasília-DF, Brazil). Paired-end whole-genome re-sequencing with 2 × 100 bp reads (CRL) and 2 × 125 bp reads (GIR, CAR, and PAN) was performed on the Illumina HiSeq2500 platform with an aimed average sequencing depth of 15X.

Pair-end reads were aligned to the *Bos taurus taurus* genome assembly UMD 3.1 using Burrows-Wheeler Alignment MEM (BWA-MEM) tool v.0.7.17 [108] and converted into a binary format using SAMtools v.1.8 [109]. Polymerase chain reaction (PCR) duplicates were marked using Picard tools (http://picard.sourceforge.net, v.2.18.2). For downstream processing, GATK v.4.0.10.1 [110–112] software was used. Base quality score recalibration was performed using a SNP database (dbSNP Build 150) retrieved from the NCBI [113] followed by SNP calling using the HaplotypeCaller algorithm. To remove unreliable SNP calls and reduce the false discovery rate, hard filtering steps were applied on the variant call. Insertions and deletions polymorphism (Indels) and multi-allelic SNPs were filtered out, and then hard filtering was applied for clustered SNPs (> 5 SNPs) in a window size of 20 bp. An outlier approach was used and values above 14.44 (highest 5%) for Fisher strand test were removed. The same was applied for the highest and lowest 2.5% values for base quality rank sum test (− 2.26 and 3.04), mapping quality rank sum test (− 2.46 and 1.58), read position rank sum test (− 1.64 and 2.18), and read depth (267 and 883). Variants with a mapping quality value lower than 30 (0.1% error probability) were also removed from the call set. SNPs that passed the filtering process and located on autosomal chromosomes were retained for subsequent analysis.

### Variant annotation and predicted functional impacts

A functional annotation analysis of the called variants was performed to assess their possible biological impact using the Variant Effect Predictor (VEP, [114]) together with the Ensembl cow gene set 94 release. Variants are categorized according to their consequence impact on protein sequence as high, moderate, low, or modifier (more severe to less severe). Variants with high consequence on protein sequence (i.e. splice acceptor variant, splice donor variant, stop gained, frameshift variant, stop lost, and start lost) were selected for further assessment. The impact of amino acid substitutions on protein function were predicted using the sorting intolerant from tolerant (SIFT) scores implemented on VEP tool, and variants with SIFT scores lower than 0.05 were considered as deleterious to protein function.

Database for Annotation, Visualization, and Integrated Discovery (DAVID) v6.8 tool [115, 116] was used to identify overrepresented GO terms and KEGG pathways using the list of genes retrieved from the variants classified with high consequence on protein sequence and as deleterious, and the *Bos taurus taurus* annotation file as a background. The *p*-values were adjusted by False Discovery Rate [21], and significant terms and pathways were considered when *p* < 0.01.

### Population differentiation analysis

A PCA implemented with a custom R script was used to examine the genetic structure of the four breeds. AMOVA [117] was also implemented to test for genetic differentiation among breeds. Such method consists in assessing population differentiation using molecular markers together with a pairwise distance matrix, and it can easily incorporate additional hierarchical levels of population structure. AMOVA computations were conducted using the ‘amova’ function in R package pegas [118]. The analyses were based on pairwise squared Euclidean distances using the ‘dist’ function implemented in R [119] and the statistical significances were tested by permutations (*n* = 1000). Additionally, the software ADMIXTURE v1.3 [120] was used to reveal admixture patterns among breeds by measuring the proportion of individual ancestry from different numbers of hypothetical ancestral populations (K). Linkage disequilibrium (LD) pruning for admixture analysis was performed on PLINK v1.90 software [121] to remove SNP with a R^2^ value greater than 0.1 with any other SNP within a 50-SNP sliding window. The optimal number of *K* was defined based on the cross-validation error value (*K* = 1 to 5) implemented in ADMIXTURE.

### Genomic inbreeding coefficient estimation

Genomic inbreeding coefficients based on runs of homozygosity (F_ROH_) were estimated for every animal according to the genome autozygotic proportion described by McQuillan et al. [122]:

$$ {F}_{ROH}^i=\frac{S_{ROH}^i}{L_{GEN}} $$

where ${S}_{ROH}^i$ is the sum of ROH across the genome for the *i*^th^ animals and *L*_*GEN*_ is the total length of the autosomes covered by SNPs. *L*_*GEN*_ was taken to be 2511.4 Mb based on the *Bos taurus taurus* genome assembly UMD 3.1. ROH were identified in every individual using PLINK v1.90 [121] software in non-overlapping sliding windows of 50 SNPs. The minimum length of a ROH was set to 500 kb. A maximum of three SNPs with missing genotypes and three heterozygous SNPs were admitted in each window, as discussed by Ceballos et al. [123]. Tukey’s post-hoc test [124] was used to identify significant pairwise comparisons (*p* < 0.05).

### Selective sweeps detection

Four statistical methods were implemented to detect genomic regions under selective pressure. Cross-population methods encompassed the Wright’s fixation index (F_ST_) and the Cross-Population Extended Haplotype Homozygosity (XPEHH). Within-population methods included the Composite Likelihood Ratio (CLR) statistic and the integrated Haplotype Score (iHS).

F_ST_ [125] was calculated between all six pairwise combinations of the four breeds with custom R scripts as follows:

$$ FST=\frac{\overline{p}\left(1-\overline{p}\right)-\sum {c}_i{p}_i\left(1-{p}_i\right)}{\overline{p}\left(1-\overline{p}\right)} $$

where $\overline{p}$ is is the average frequency of an allele in the total population, *p*_*i*_ is the allele frequency in the i^*th*^ population, and *c*_*i*_ is the relative number of SNPs in the i^*th*^ population. F_ST_ scores were then averaged in non-overlapping sliding windows of 50 kb. SweepFinder2 software [126] was used to calculate the CLR statistic [127] within each breed in non-overlapping sliding windows of 50 kb across the genome. The ancestral allele information was assessed from a cattle reference allele list retrieved from Rocha et al. [128]. The CLR analysis was performed considering only SNPs containing the ancestral allele information (*n =* 11,260,629 SNPs). The iHS [129] and XP-EHH [130] statistics were calculated using the program selscan v1.2.0a [131] with default parameters. Within each population, haplotype phasing was performed using Beagle 5.0 [132] and the genetic distances were determined by assuming that 1 Mb ≈ 1 centiMorgan (cM). The iHS scores were calculated within each breed and XP-EHH between all six pairwise combinations of the four breeds. The unstandardized iHS and XP-EHH scores were standard normalized using the script norm with default parameters, as provided by selscan. Absolute iHS and XP-EHH values were averaged in non-overlapping sliding windows of 50 kb. To compute the iHS statistic, the same subset of SNPs (*n =* 11,260,629 SNPs) applied in the CLR statistic was used, however, without considering any ancestral allele information. Independent results for each statistical method and population implemented herein are presented in Additional file 13.

Selective sweeps detection can be enhanced by combining multiple genome-wide scan methodologies, benefiting from advantageous complementarities among them together with the increase in the statistical power [20, 133–136]. Further, combining within-population statistics from multiple breeds may decrease false-positive signals that arise due to population stratification (reviewed by Hellwege et al. [137]). Accordingly, within-population and cross-population statistics were combined separately in a single score using the DCMS statistic [20]. The DCMS statistic was calculated for each 50 kb window using the MINOTAUR package [138] and the empirical *p*-values of each statistic were derived from a skewness normal distribution with an appropriate one-tailed test (Additional file 14). Candidate sweep regions under selection were revealed by assessing the top 1% of the empirical distribution generated by the DCMS statistics.

Candidate regions identified herein were compared with previous regions under selection described in the literature for other cattle breeds. Overlap analysis was carried out using the Bioconductor package *GenomicRanges* [139].

### Selective sweeps and runs of homozygosity

Candidate sweep regions revealed from the top 1% of the empirical distribution generated by the DCMS statistics were intersected with ROH hotspots to identify common signals between both methodologies. ROH formerly identified to estimate F_ROH_ were applied, and ROH hotspots were determined by selecting segments shared by more than 50% of the samples within each breed.

Overlap analysis was performed separately for each DCMS statistic using the Bioconductor package *GenomicRanges* [139].

### Functional annotation of the candidate regions

Genes were annotated within the candidate sweep regions using the cow gene set Ensembl release 94 fetched from the Biomart tool [140]. BEDTools [141] was used to identify overlaps between the retrieved gene set list and the putative sweep regions. DAVID v6.8 tool [115, 116] was used to identify overrepresented GO terms and KEGG pathways using the list of genes from the putative sweep regions and the *Bos taurus taurus* annotation file as a background. The *p*-values were adjusted by False Discovery Rate [21], and significant terms and pathways were considered when *p* < 0.01. QTLs retrieved from the CattleQTL database [142] were overlapped with the candidate sweep regions using BEDtools [141].

## Supplementary information

**Additional file 1. **Distribution of the functional consequences of the called variants (*n* = 33,328,447 SNPs) using the Variant Effect Predictor (VEP) tool.

**Additional file 2. **Gene Ontology (GO) terms and Kyoto Encyclopedia of Genes and Genomes (KEGG) pathways analysis enriched (*p* < 0.01) based on variants with high consequence on protein sequence set of genes

**Additional file 3.** Gene Ontology (GO) terms and Kyoto Encyclopedia of Genes and Genomes (KEGG) pathways analysis enriched (p < 0.01) based on deleterious variants (SIFT score < 0.05) set of genes

**Additional file 4.** Analysis of Molecular Variance results.

**Additional file 5.** Annotated candidate sweep regions for the within-population statistic retrieved from the top 1% of the empirical distribution generated by the DCMS statistic.

**Additional file 6.** Annotated candidate sweep regions for the cross-population statistic retrieved from the top 1% of the empirical distribution generated by the DCMS statistic.

**Additional file 7.** Runs of homozygosity (ROH) hotspots for Gir (GIR), Caracu Caldeano (CAR), Crioulo Lageano (CRL), and Pantaneiro (PAN) cattle breeds.

**Additional file 8.** Overlapping of the putative sweep regions identified from the top 1% of the within-population DCMS statistic with candidate regions under positive selection previously reported in other cattle populations.

**Additional file 9.** Overlapping of the putative sweep regions identified from the top 1% of the cross-population DCMS statistic with candidate regions under positive selection previously reported in other cattle populations.

**Additional file 10.** Overlapping between ROH hotspots and the top 1% of the within-population DCMS statistic with the candidate regions under positive selection previously reported in other cattle populations.

**Additional file 11.** Overlapping between ROH hotspots and the top 1% of the cross-population DCMS statistic with the candidate regions under positive selection previously reported in other cattle populations.

**Additional file 12.** Brazilian geographical regions of the four cattle breeds sampled in the study (Adapted from https://pt.wikipedia.org/wiki/Ficheiro:Brazil_Labelled_Map.svg).

**Additional file 13.** Manhattan plot of the independent results for each selective sweep statistical method and population.

**Additional file 14.** Histogram and quantile-quantile (Q-Q) plots of statistical scores calculated for all four methods derived from a skewness normal distribution.

## Data Availability

The genomic information used in this study is available from EMBRAPA – Brazilian Agriculture Research Corporation (EMBRAPA SEG 20.18.01.018.00.00), but restrictions apply to their public availability. However, data are available for sharing upon reasonable request and with permission of the corresponding author Marcos Vinícius Gualberto Barbosa da Silva, e-mail: marcos.vb.silva@embrapa.br The *Bos taurus taurus* genome assembly (UMD 3.1) used in this study can be found in https://www.ncbi.nlm.nih.gov/assembly/GCF_000003055.4/ (RefSeq assembly accession: GCF_000003055.4). The SNP database (dbSNP Build 150) used in this study can be found in https://ftp.ncbi.nih.gov/snp/organisms/archive/cow_9913/VCF/. Overrepresented GO terms and KEGG pathways described in this study were retrieved from DAVID (Database for Annotation, Visualization, and Integrated Discovery) v6.8 tool [115, 116].

## Abbreviations

DCMS: De-correlated composite of multiple signals
RAPD: Random amplified polymorphic DNA
SNP: Single-nucleotide polymorphism
GIR: Gir
CAR: Caracu Caldeano
CRL: Crioulo Lageano
PAN: Pantaneiro
AMOVA: Analysis of molecular variance
K: Proportions of ancestry
F_ROH_: Runs of homozygosity-based inbreeding coefficients
BTA: *Bos taurus* autosome
GO: Gene ontology
KEGG: Kyoto Encyclopedia of Genes and Genomes
QTL: Quantitative trait locus
ROH: Runs of homozygosity
PCA: Principal component analysis
PNMGL: Brazilian Dairy Gir Breeding Program
ABS: American Breeders Service
CRV: Cooperatie Rundvee Verbetering
BWA-MEM: Burrows-Wheeler Alignment MEM
PCR: Polymerase chain reaction
VEP: Variant Effect Predictor
SIFT: Sorting intolerant from tolerant
DAVID: Database for Annotation, Visualization, and Integrated Discovery
LD: Linkage disequilibrium
F_ST_: Wright’s fixation index
XPEHH: Cross-population extended haplotype homozygosity
CLR: Composite likelihood ratio
iHS: integrated haplotype score
cM: centimorgan
