# Supplementary material for: Genome-wide detection of signatures of selection in indicine and Brazilian locally adapted taurine cattle breeds using whole-genome re-sequencing data
Source: BMC Genomics. 2020 Sep 11;21:624. doi: 10.1186/s12864-020-07035-6 (PMC7488563; doi:10.1186/s12864-020-07035-6)
Supplement: Supplementary file 4 — Additional file 4. Analysis of Molecular Variance results. [file 12864_2020_7035_MOESM4_ESM.docx]

**Analysis of Molecular Variance between Pantaneiro (PAN) and Crioulo Lageano (CRL) cattle breeds**

Call: pegas::amova(formula = gen_dist ~ info_factor, is.squared = TRUE)

SSD MSD df

info_factor 34.34943 34.34943 1

Error 642.59911 29.20905 22

Total 676.94853 29.43254 23

Variance components:

sigma2 P.value

info_factor 0.42836 0

Error 29.20905

Phi-statistics:

info_factor.in.GLOBAL

0.01445351

Variance coefficients:

a

12

**Analysis of Molecular Variance of the four breeds**

Call: pegas::amova(formula = gen_dist ~ Breeds, is.squared = TRUE)

SSD MSD df

Breeds 194.6287 64.87624 3

Error 1272.8329 28.28517 45

Total 1467.4616 30.57212 48

Variance components:

sigma2 P.value

Breeds 2.9883 0

Error 28.2852

Phi-statistics:

Breeds.in.GLOBAL

0.09555297

Variance coefficients:

a

122.449
