# Supplementary figures and images for: Genome-wide detection of signatures of selection in indicine and Brazilian locally adapted taurine cattle breeds using whole-genome re-sequencing data
Source: BMC Genomics. 2020 Sep 11;21:624. doi: 10.1186/s12864-020-07035-6 (PMC7488563; doi:10.1186/s12864-020-07035-6)

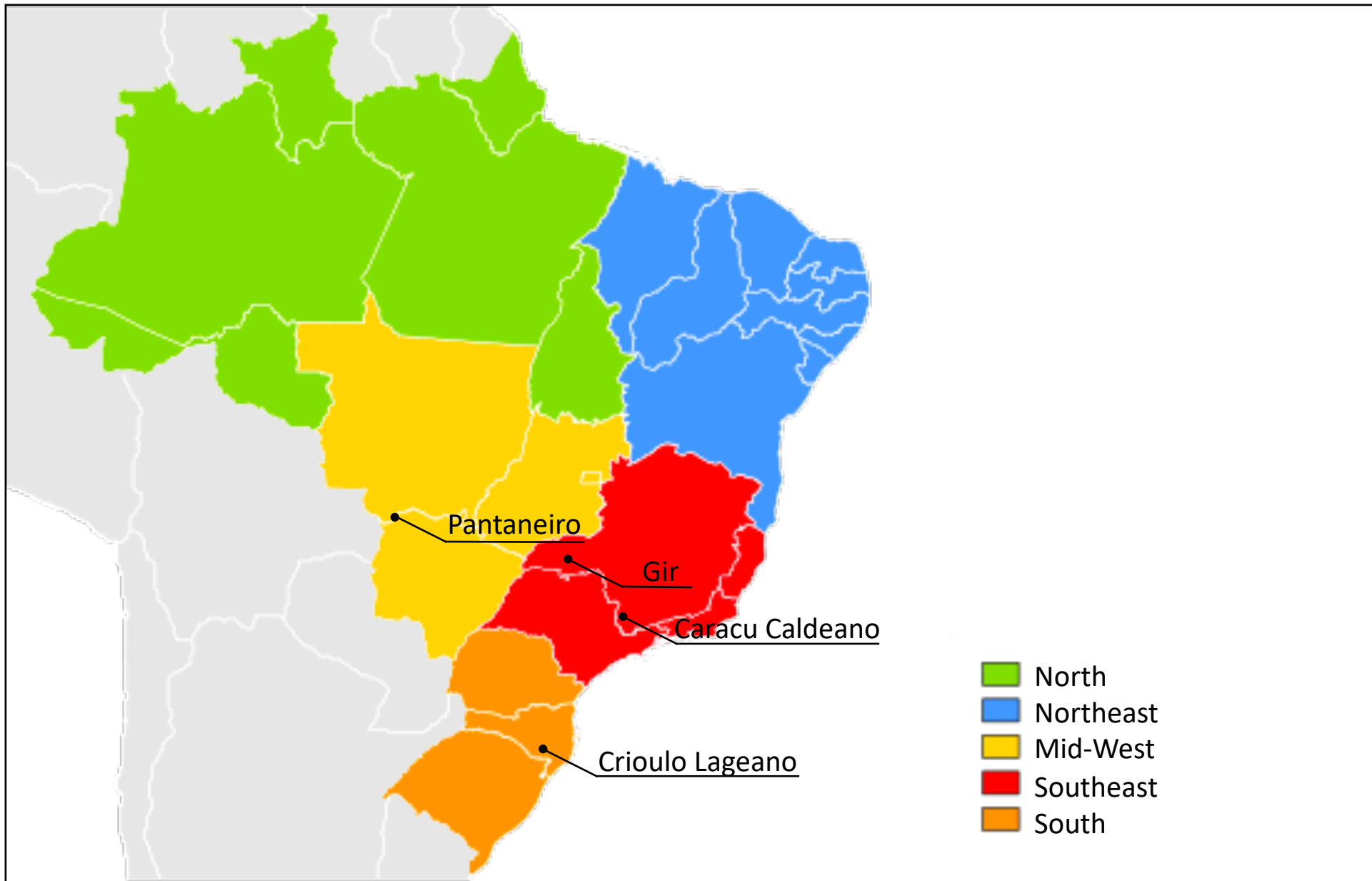

Supplement: Supplementary file 12 — Additional file 12. Brazilian geographical regions of the four cattle breeds sampled in the study (Adapted from https://pt.wikipedia.org/wiki/Ficheiro:Brazil_Labelled_Map.svg). [file 12864_2020_7035_MOESM12_ESM.pdf]

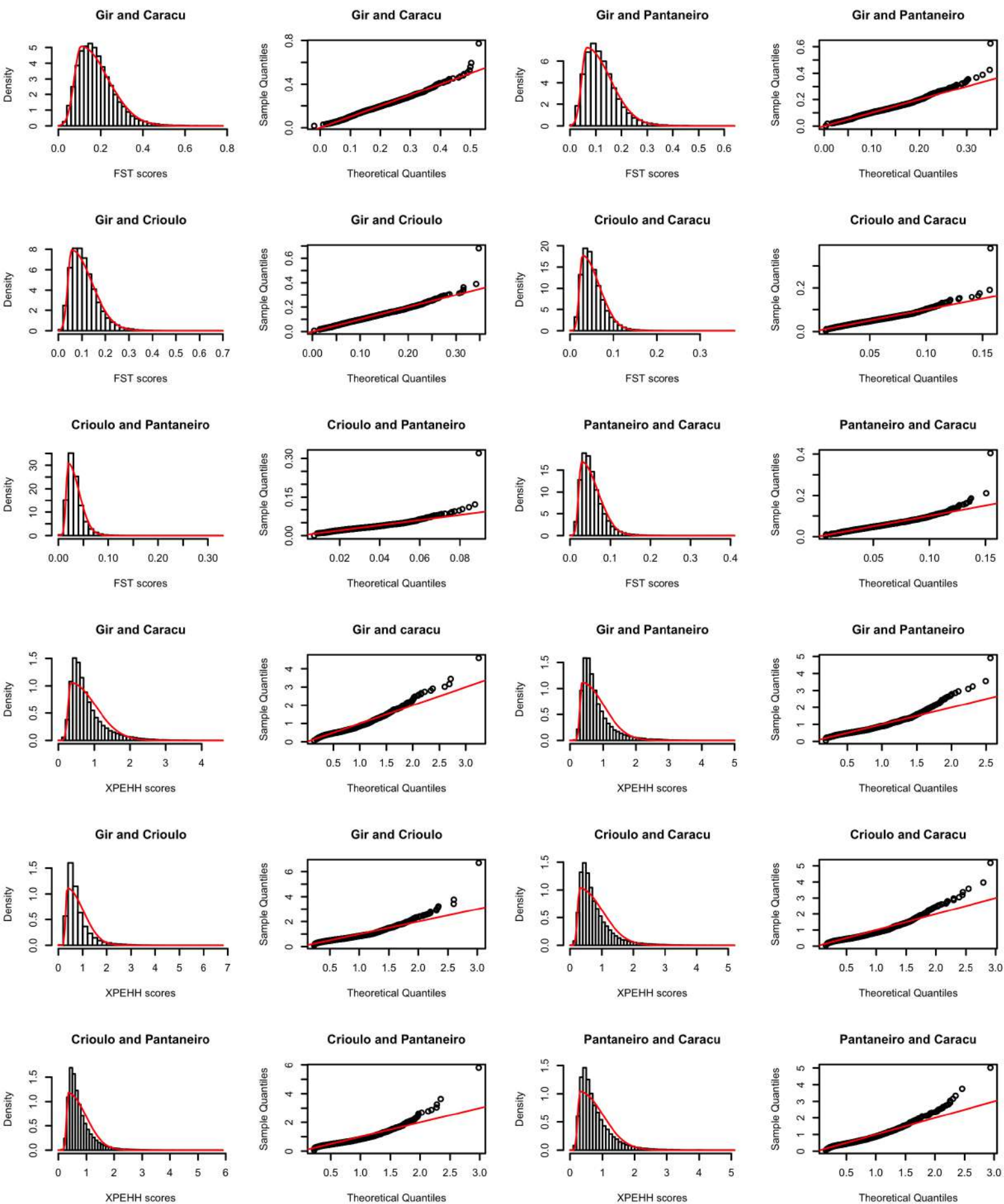

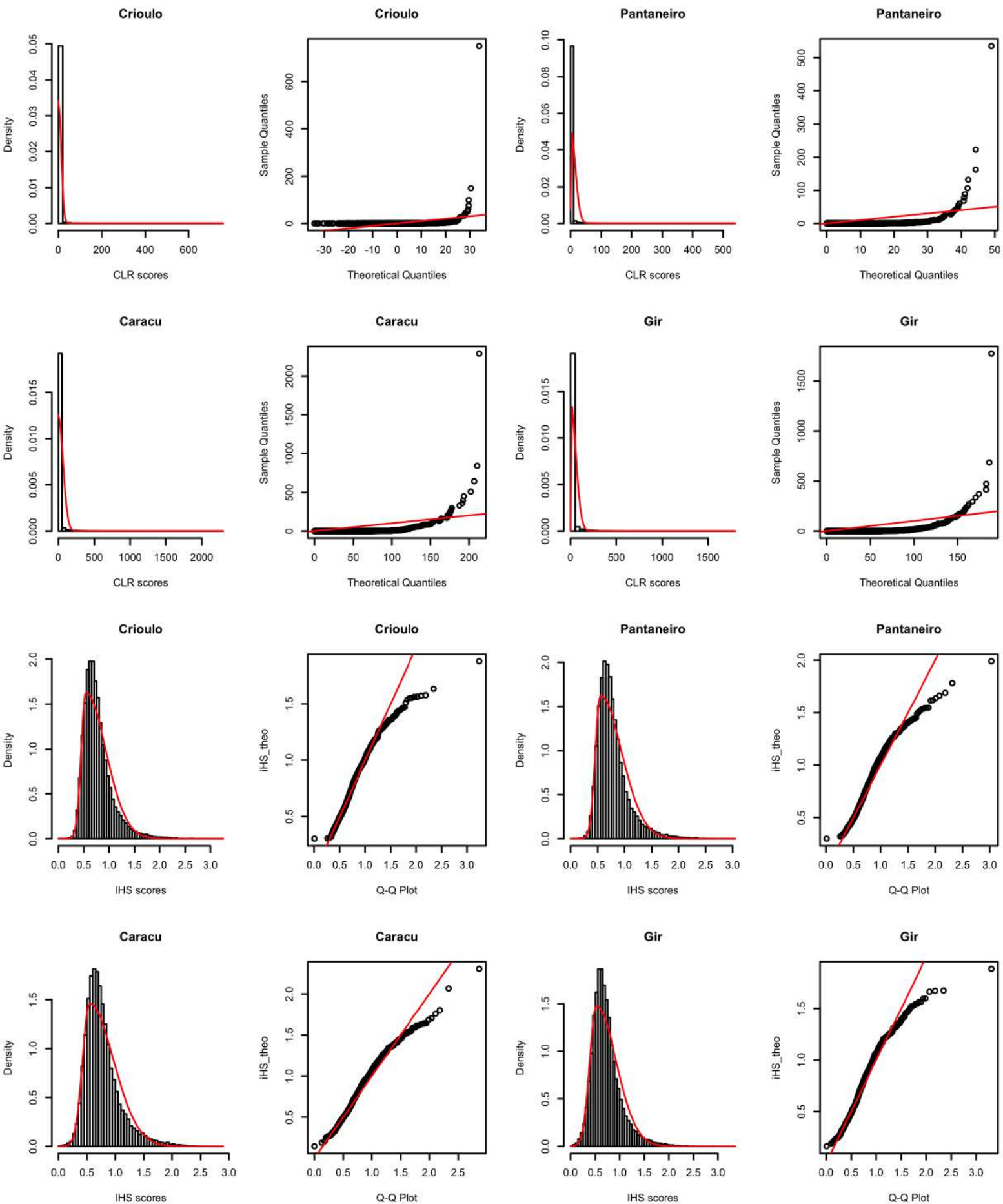

Supplement: Supplementary file 14 — Additional file 14. Histogram and quantile-quantile (Q-Q) plots of statistical scores calculated for all four methods derived from a skewness normal distribution. [file 12864_2020_7035_MOESM14_ESM.pdf]
