# Supplementary material for: Genome-wide detection of signatures of selection in indicine and Brazilian locally adapted taurine cattle breeds using whole-genome re-sequencing data
Source: BMC Genomics. 2020 Sep 11;21:624. doi: 10.1186/s12864-020-07035-6 (PMC7488563; doi:10.1186/s12864-020-07035-6)
Supplement: Supplementary file 13 — Additional file 13. Manhattan plot of the independent results for each selective sweep statistical method and population. [file 12864_2020_7035_MOESM13_ESM.pdf]

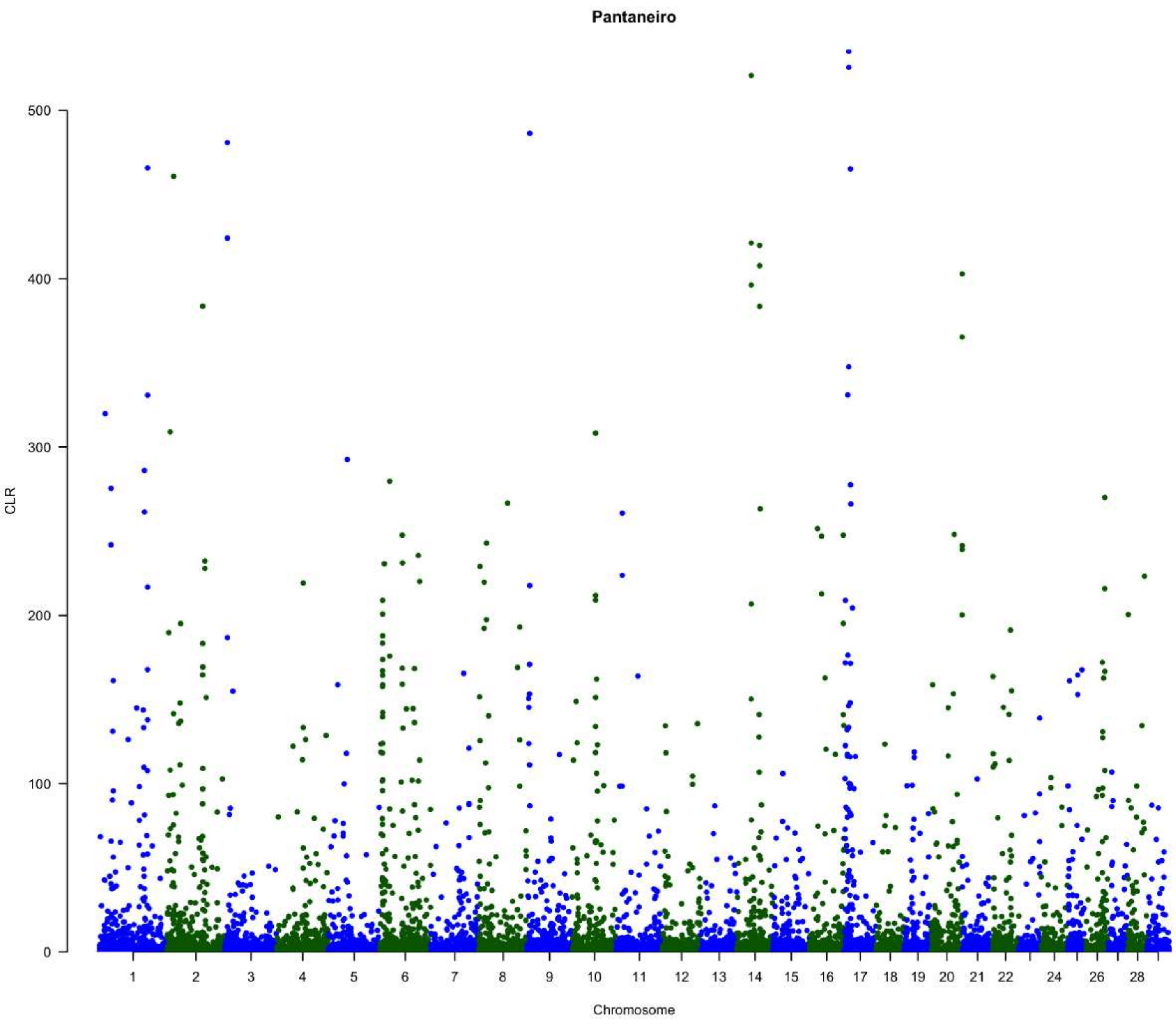
Pantaneiro
CLR
0
100
200
300
400
500
Chromosome
1
2
3
4
5
6
7
8
9
10
11
12
13
14
15
16
17
18
19
20
21
22
24
26
28

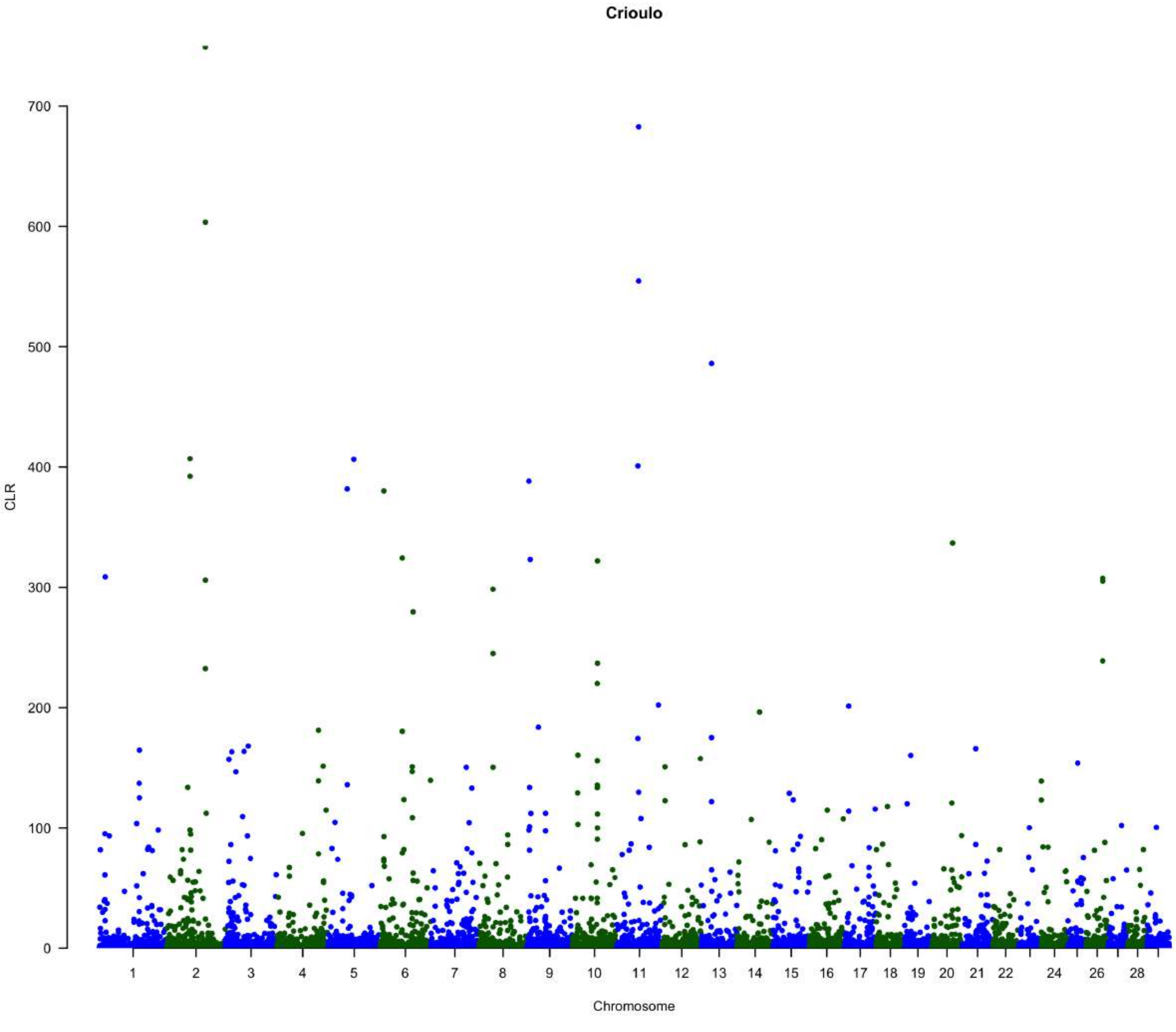

Crioulo
CLR
0
100
200
300
400
500
600
700
Chromosome
1
2
3
4
5
6
7
8
9
10
11
12
13
14
15
16
17
18
19
20
21
22
24
26
28

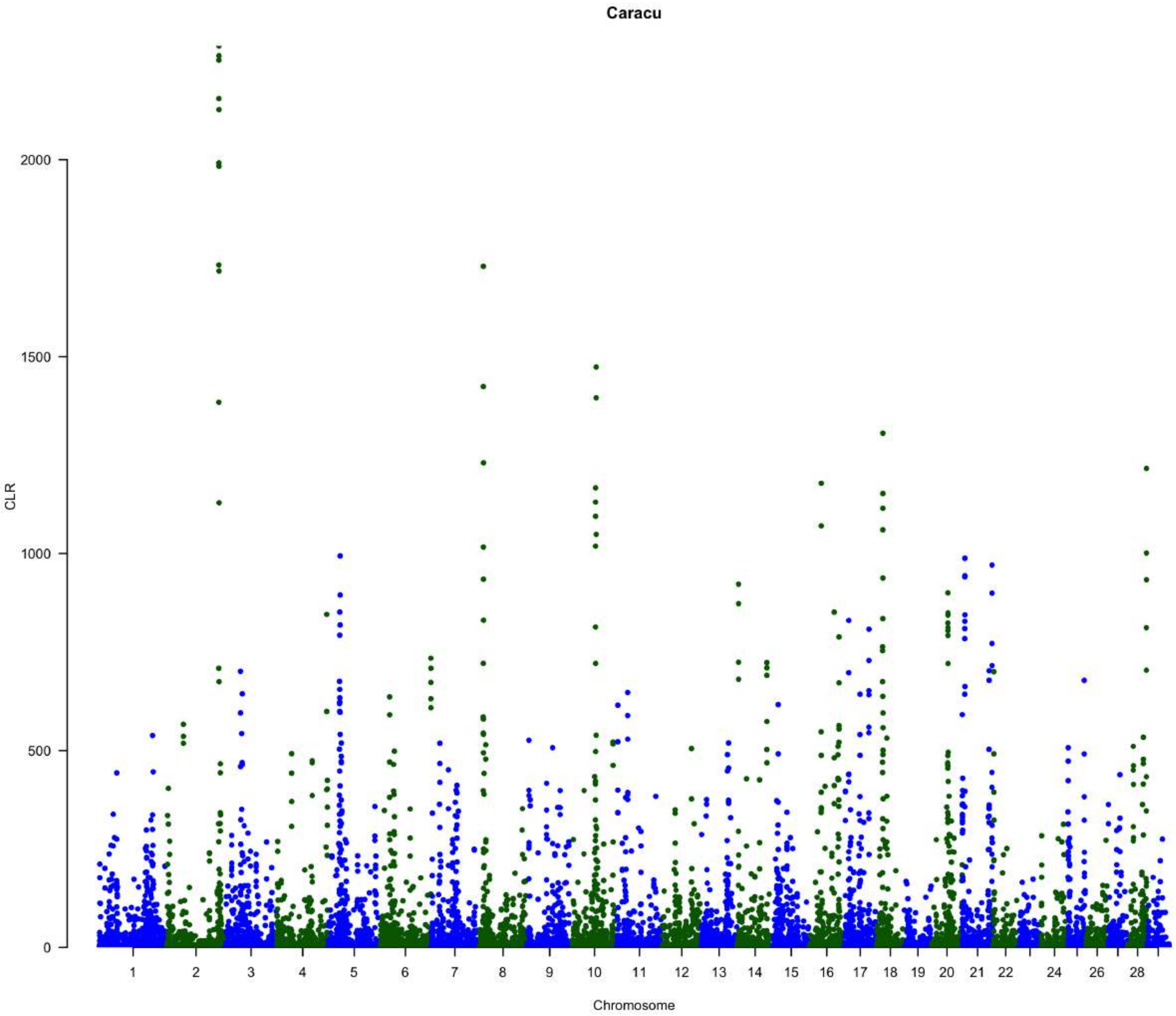

Caracu
CLR
2000
1500
1000
500
0
1
2
3
4
5
6
7
8
9
10
11
12
13
14
15
16
17
18
19
20
21
22
24
26
28
Chromosome

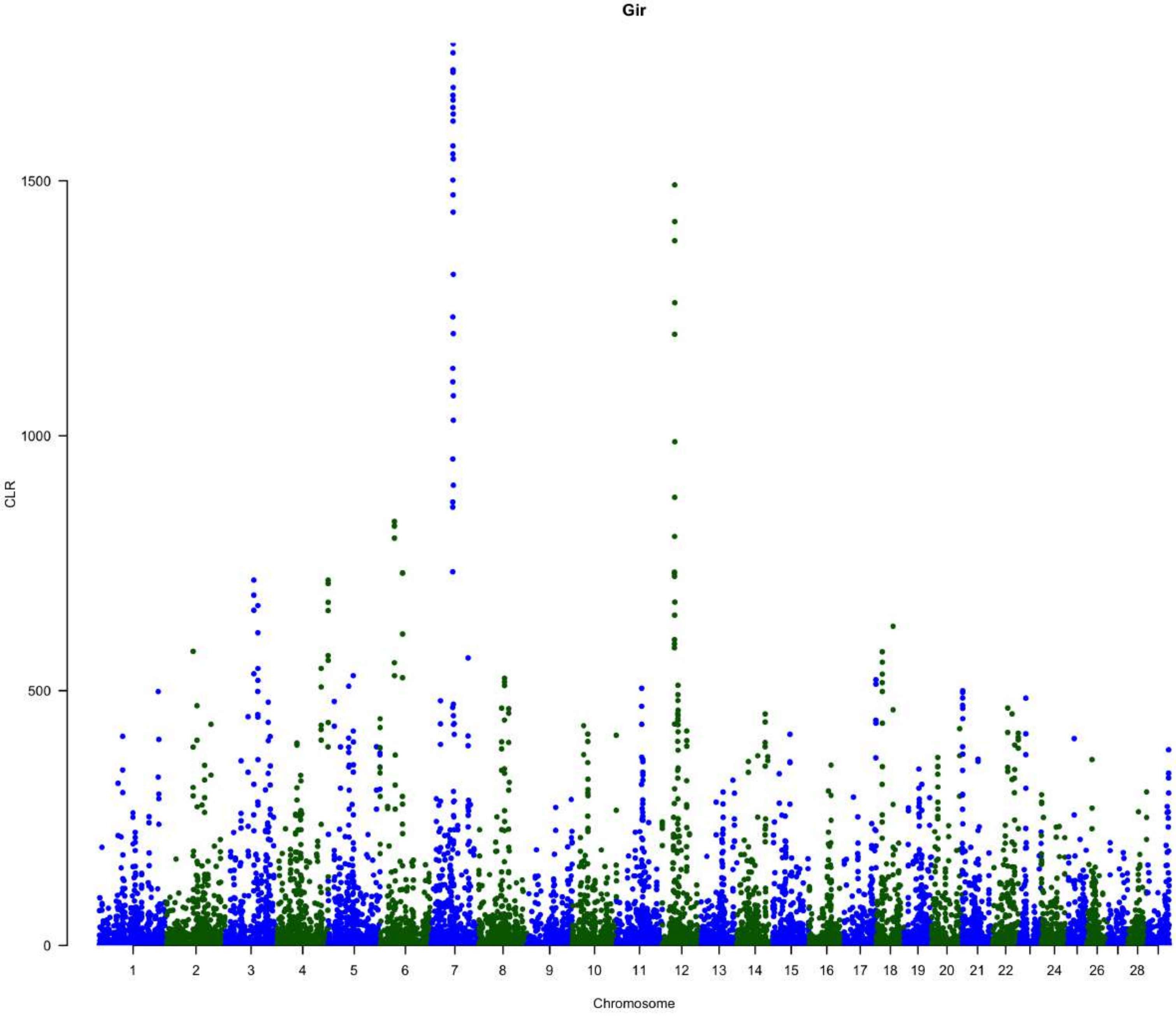
Gir
CLR
0
500
1000
1500
Chromosome
1
2
3
4
5
6
7
8
9
10
11
12
13
14
15
16
17
18
19
20
21
22
24
26
28

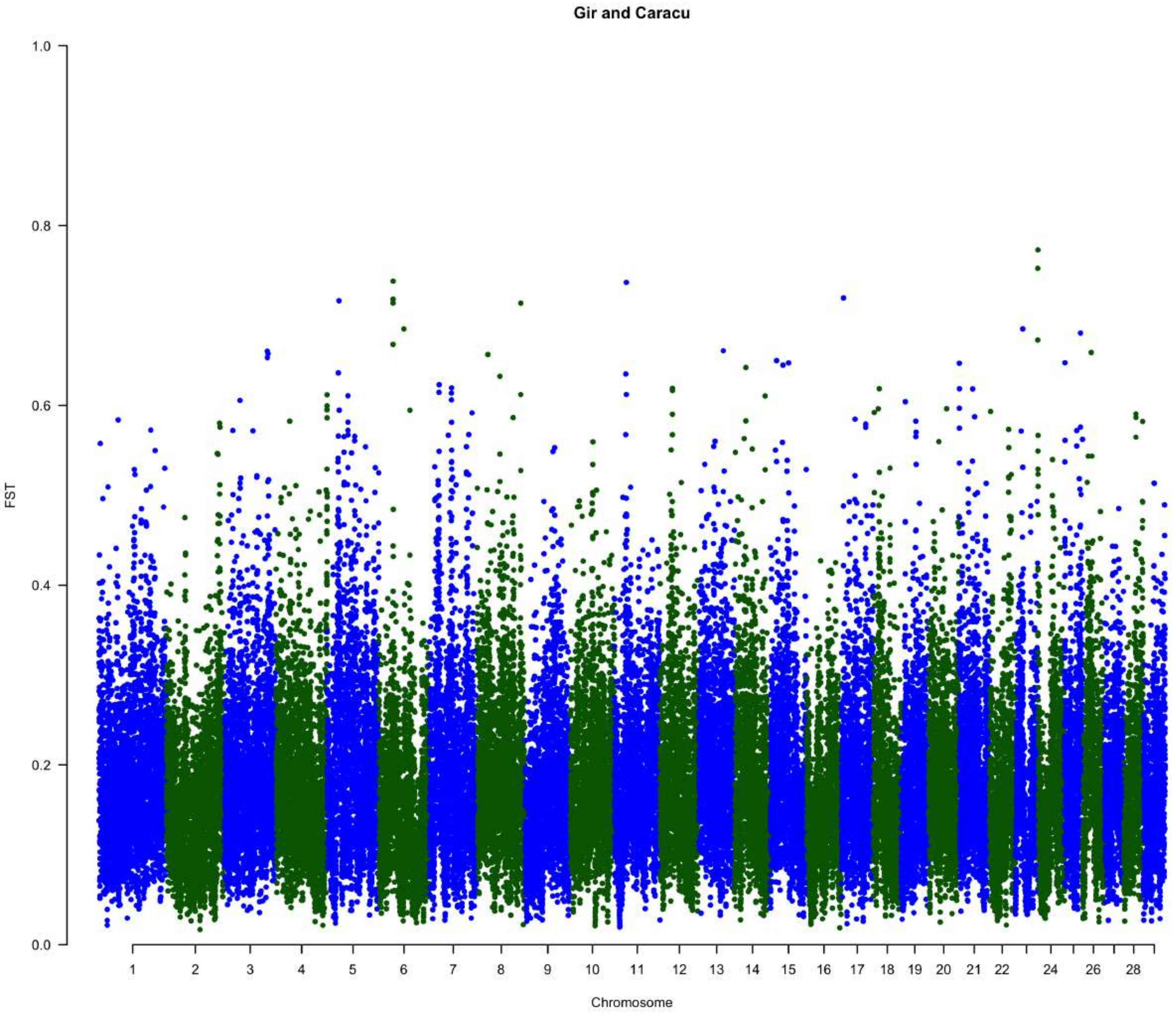
Gir and Caracu
FST
0.0
0.2
0.4
0.6
0.8
1.0
Chromosome
1
2
3
4
5
6
7
8
9
10
11
12
13
14
15
16
17
18
19
20
21
22
24
26
28

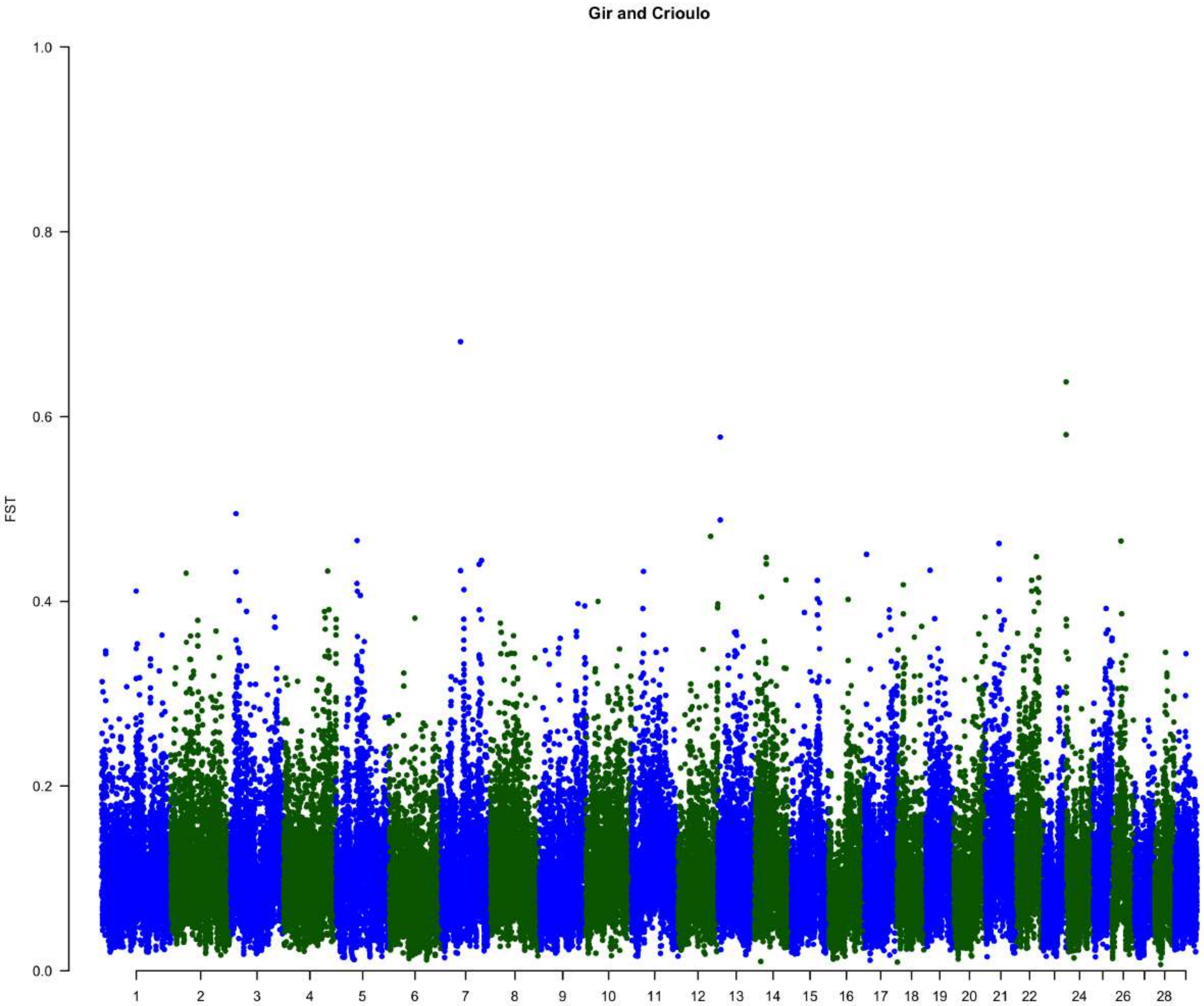

Gir and Crioulo
FST
1.0
0.8
0.6
0.4
0.2
0.0
Chromosome
1
2
3
4
5
6
7
8
9
10
11
12
13
14
15
16
17
18
19
20
21
22
24
26
28

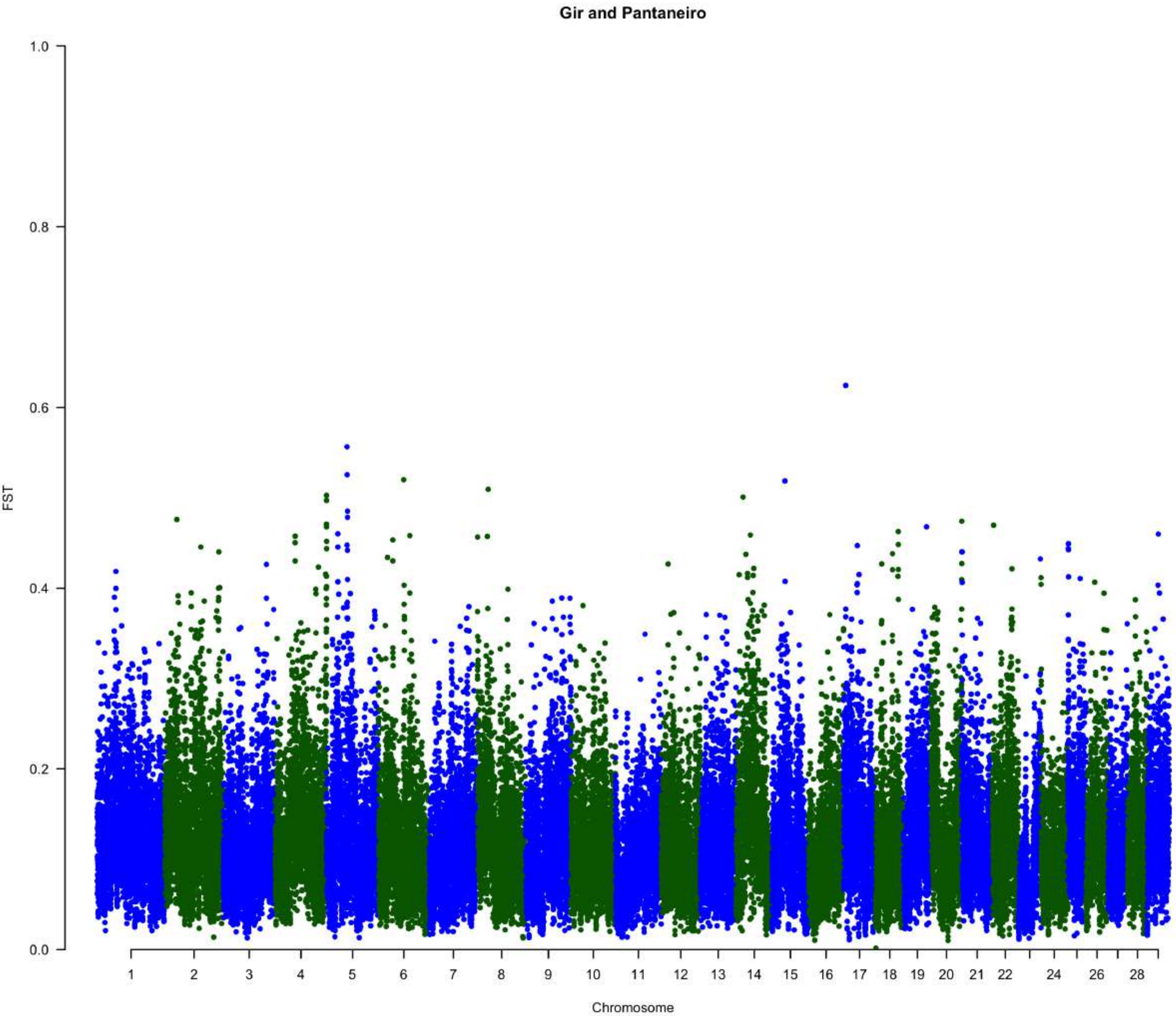

Gir and Pantaneiro
1.0
0.8
0.6
0.4
0.2
0.0
FST
1
2
3
4
5
6
7
8
9
10
11
12
13
14
15
16
17
18
19
20
21
22
24
26
28
Chromosome

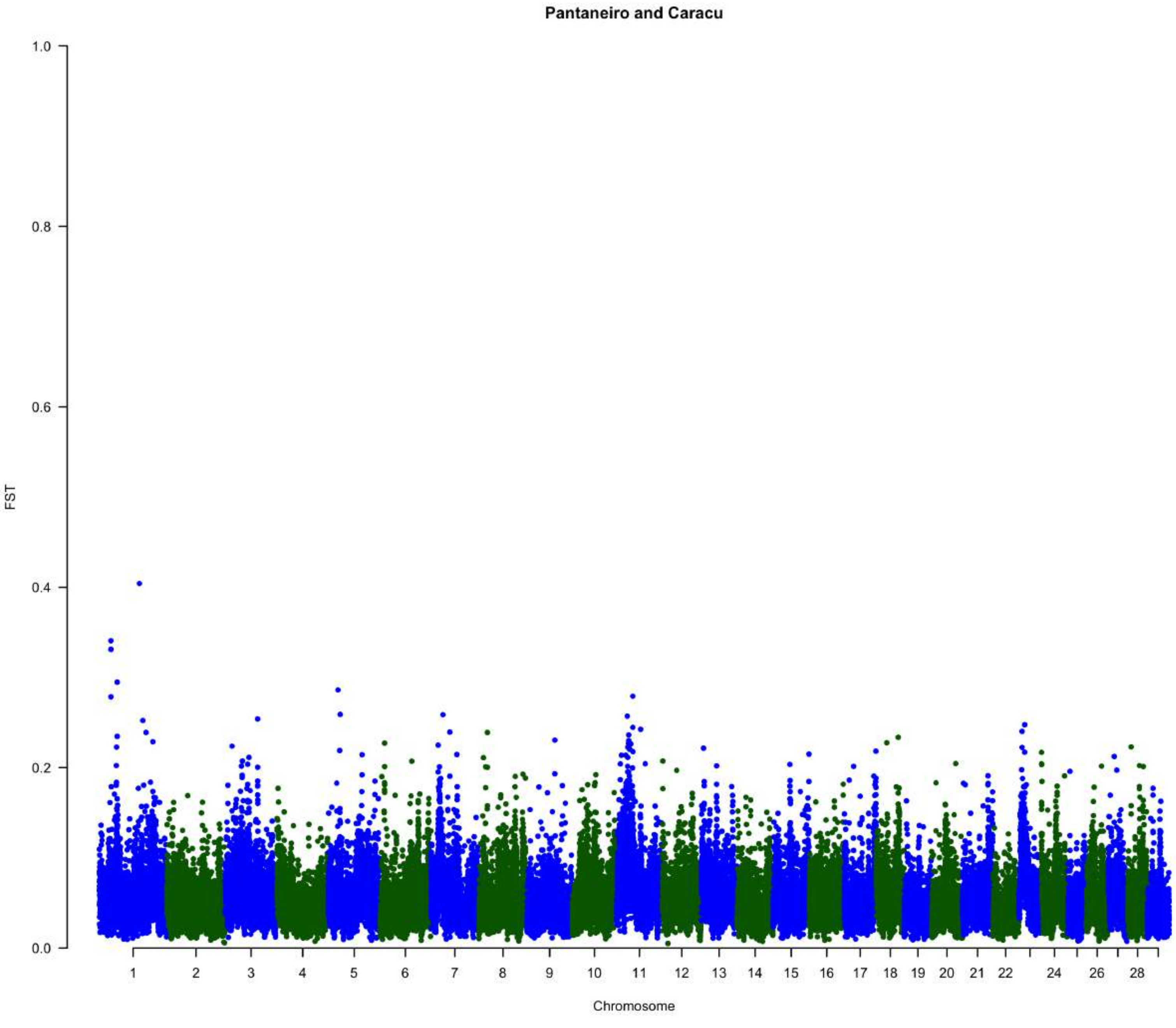
Pantaneiro and Caracu
FST
1.0
0.8
0.6
0.4
0.2
0.0
1
2
3
4
5
6
7
8
9
10
11
12
13
14
15
16
17
18
19
20
21
22
24
26
28
Chromosome

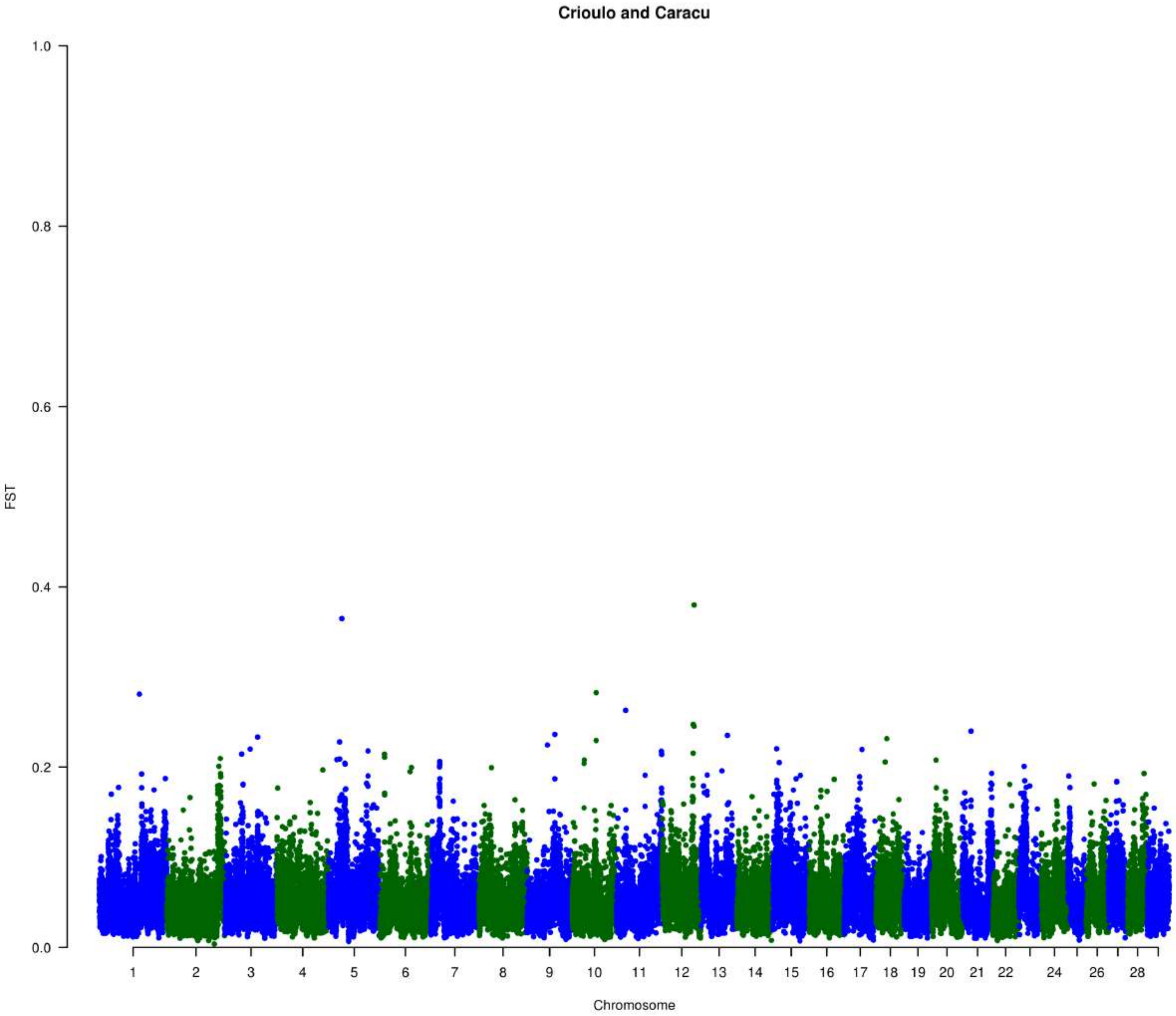
Crioulo and Caracu
FST
1.0
0.8
0.6
0.4
0.2
0.0
1 2 3 4 5 6 7 8 9 10 11 12 13 14 15 16 17 18 19 20 21 22 24 26 28
Chromosome

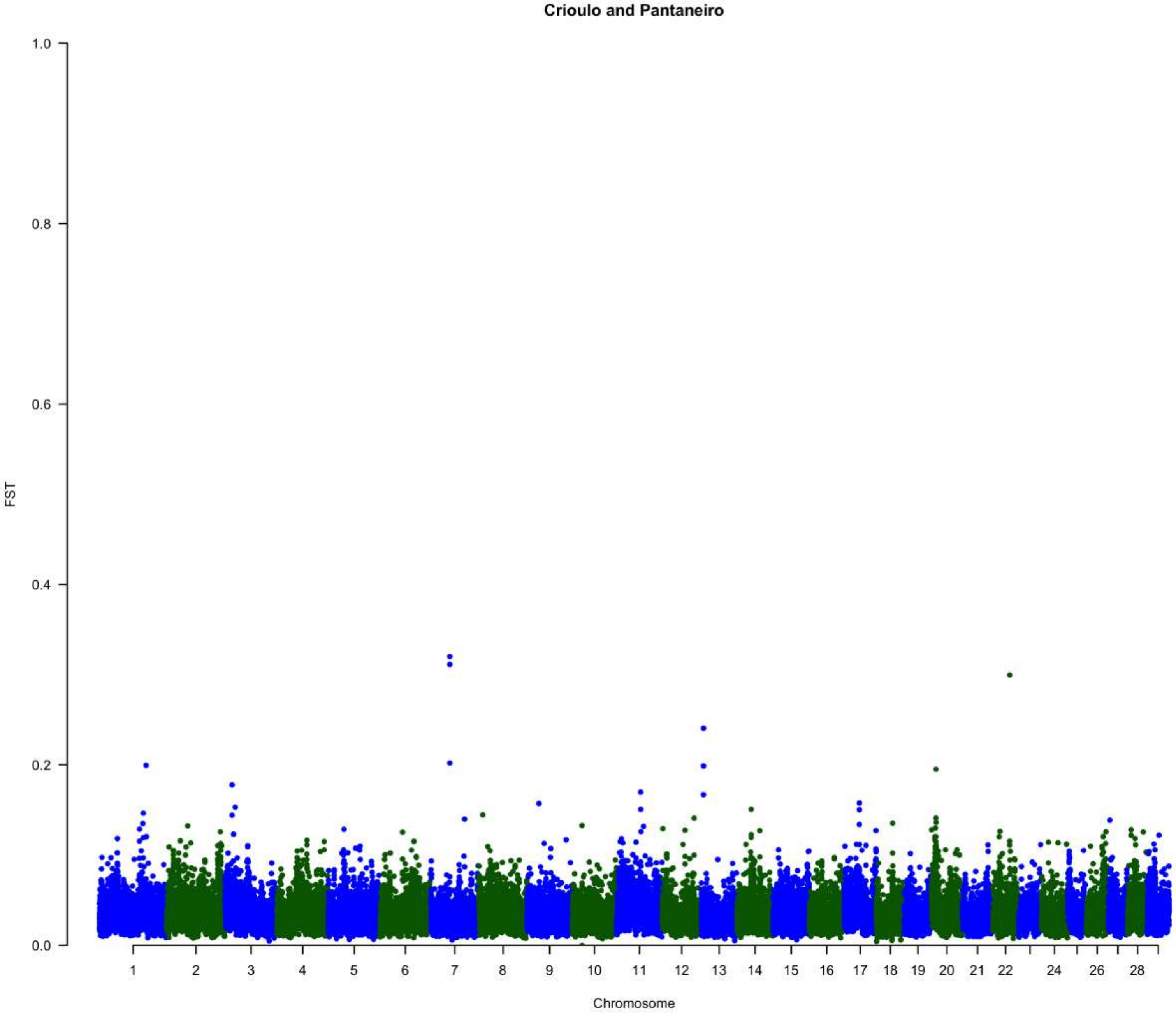

Crioulo and Pantaneiro
FST
1.0
0.8
0.6
0.4
0.2
0.0
1
2
3
4
5
6
7
8
9
10
11
12
13
14
15
16
17
18
19
20
21
22
24
26
28
Chromosome

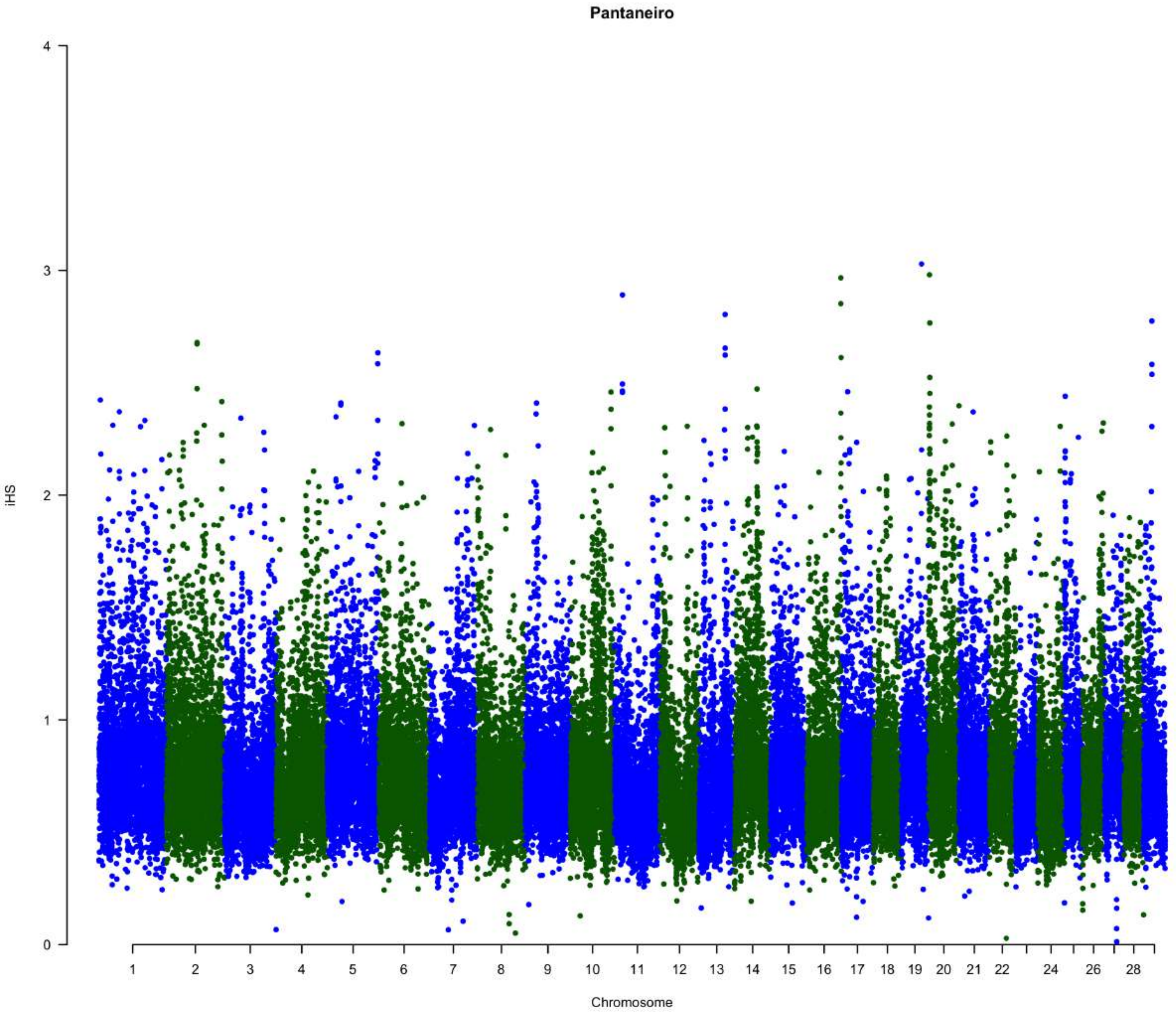

Pantaneiro
iHS
0
1
2
3
4
Chromosome
1
2
3
4
5
6
7
8
9
10
11
12
13
14
15
16
17
18
19
20
21
22
24
26
28

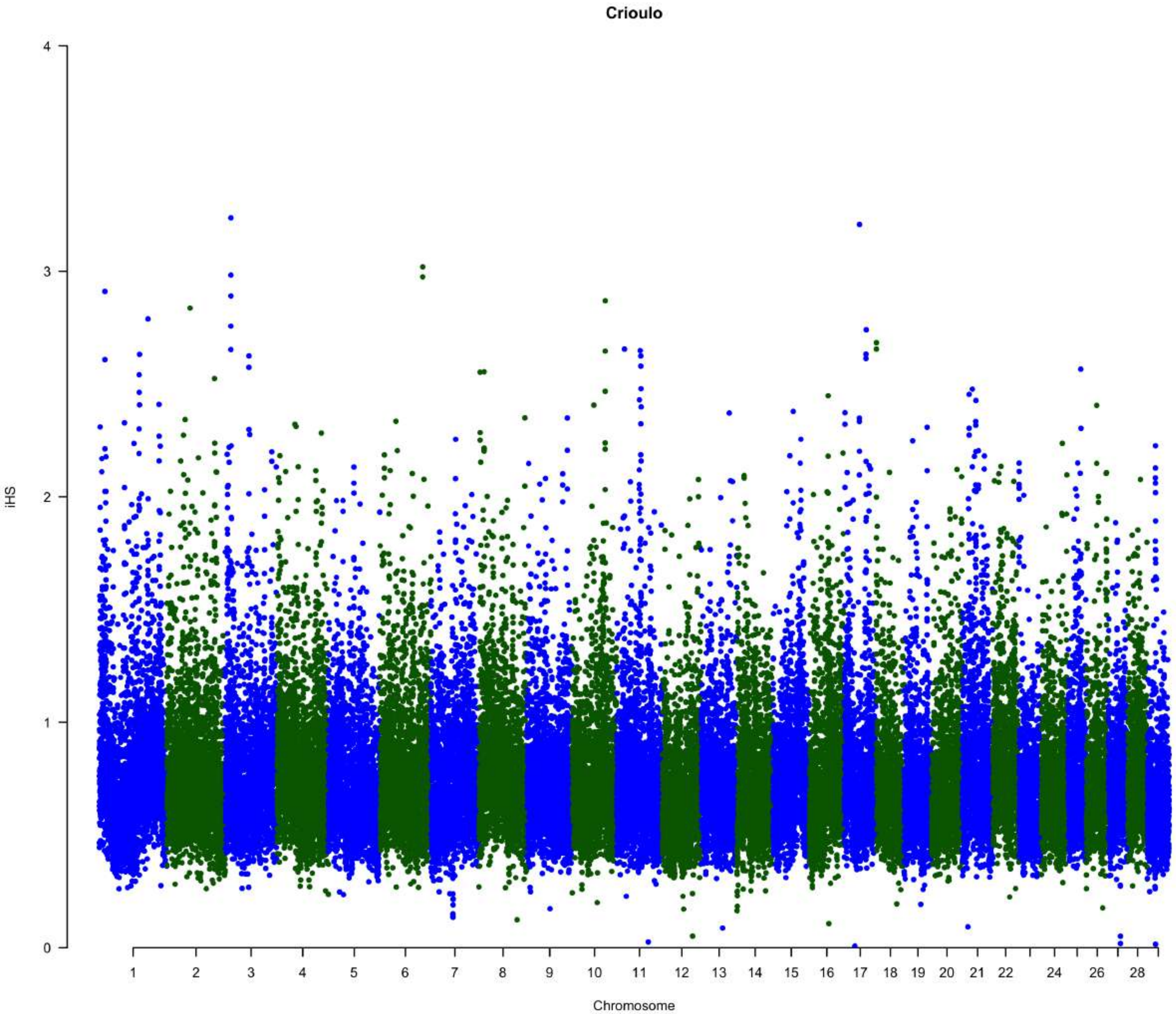
Crioulo
iHS
Chromosome
0
1
2
3
4
1
2
3
4
5
6
7
8
9
10
11
12
13
14
15
16
17
18
19
20
21
22
24
26
28

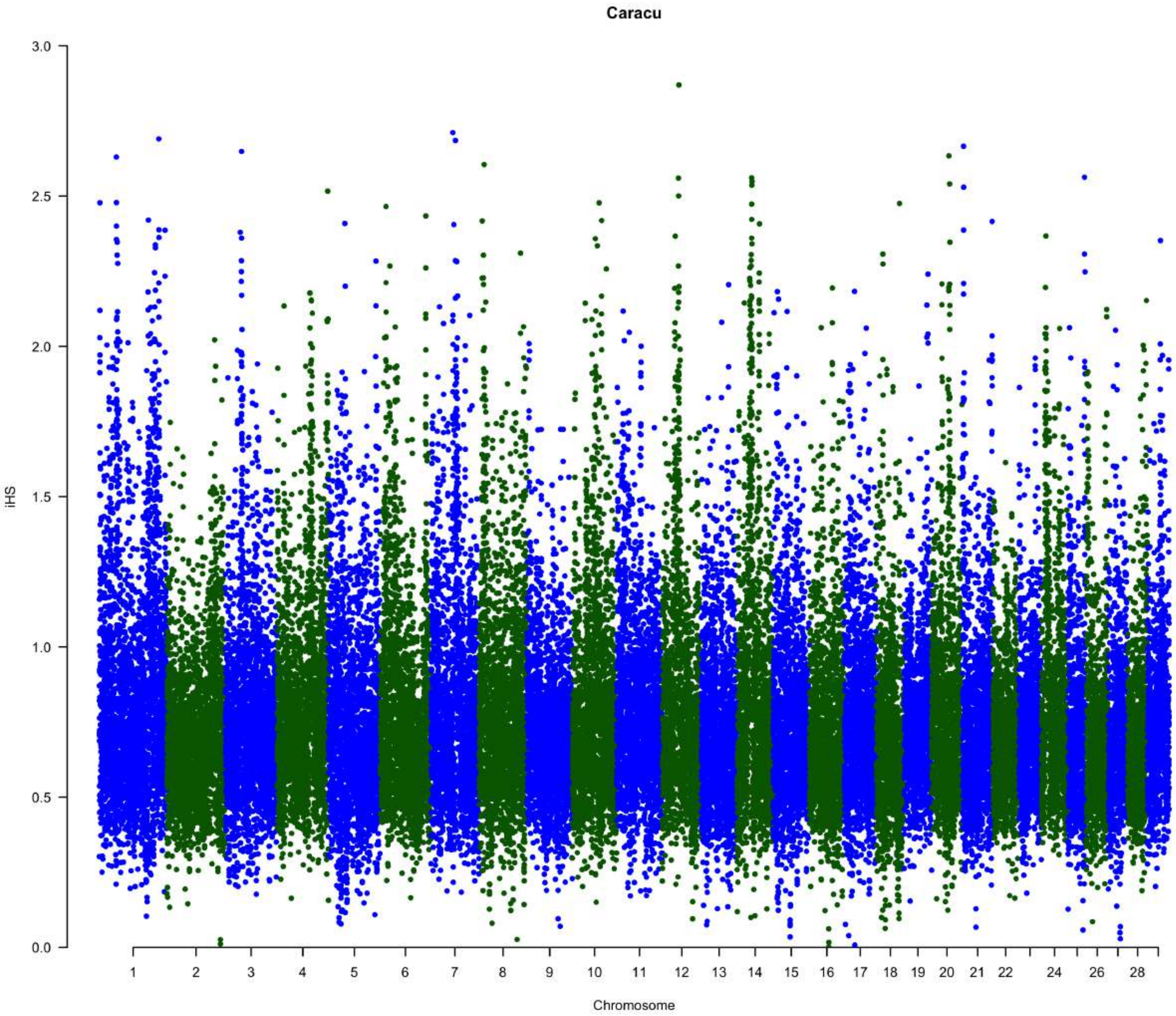
Caracu
iHS
Chromosome
0.0
0.5
1.0
1.5
2.0
2.5
3.0
1
2
3
4
5
6
7
8
9
10
11
12
13
14
15
16
17
18
19
20
21
22
24
26
28

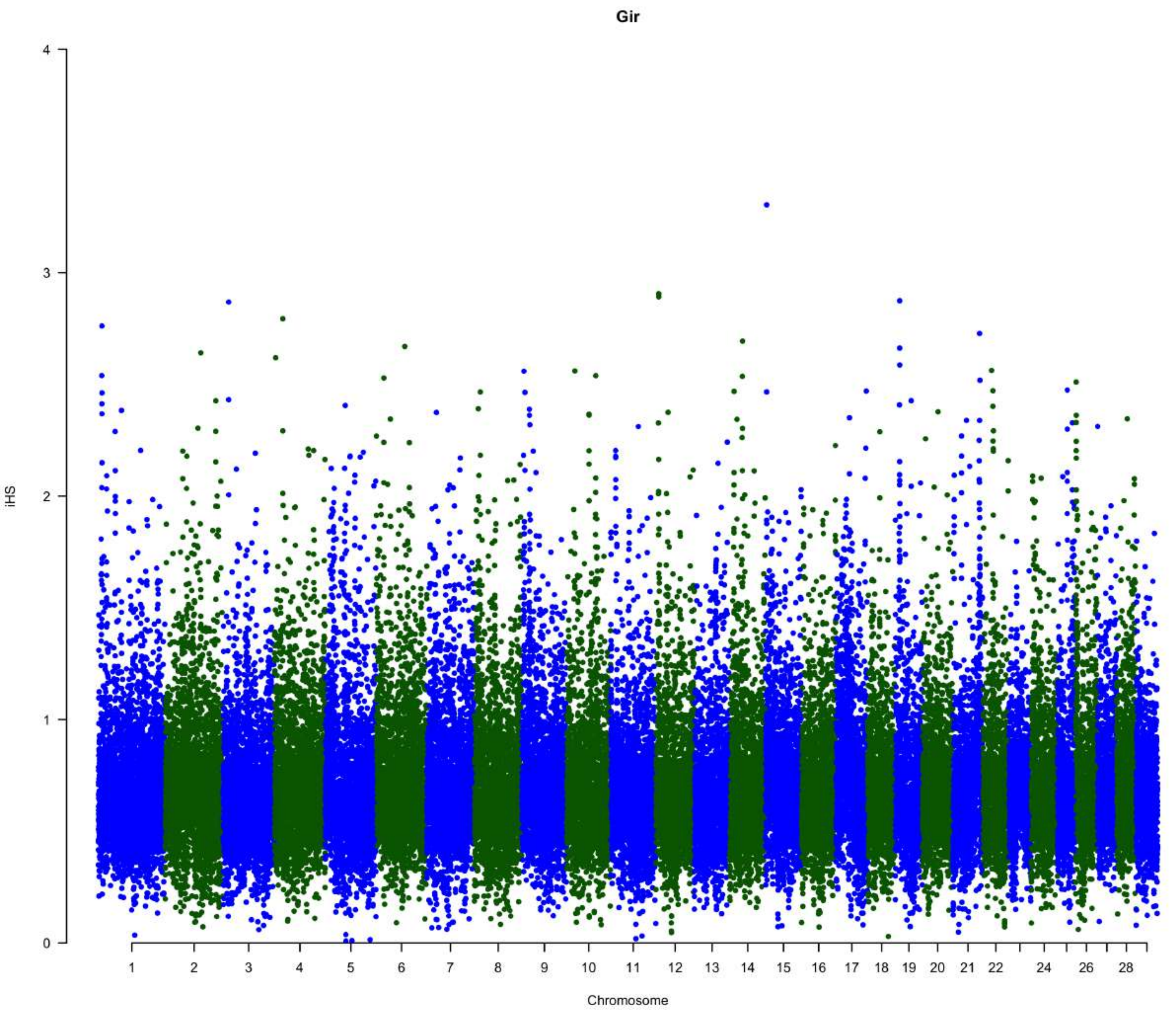

Gir
iHS
Chromosome
0
1
2
3
4
1
2
3
4
5
6
7
8
9
10
11
12
13
14
15
16
17
18
19
20
21
22
24
26
28

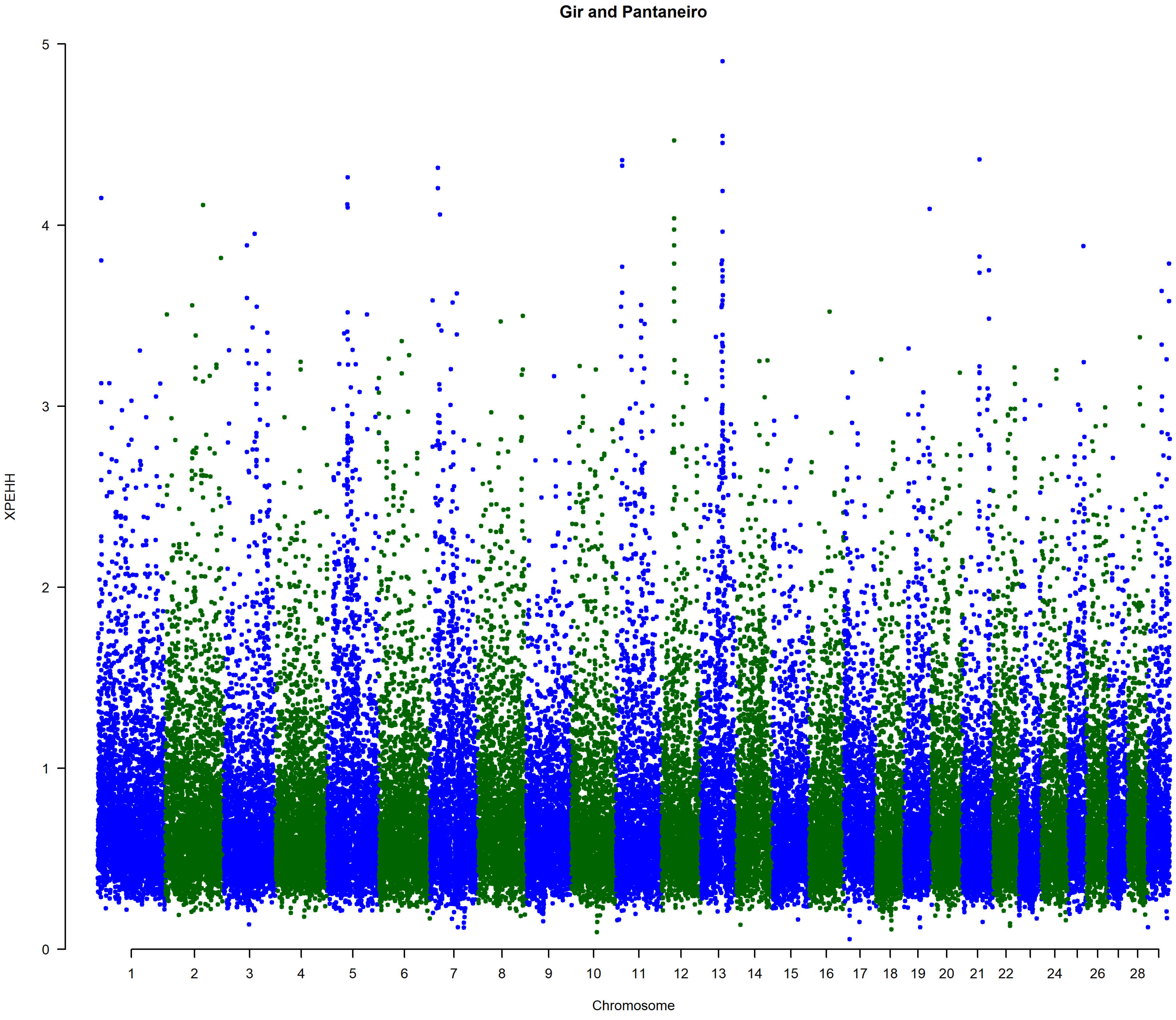

Gir and Pantaneiro
XPEHH
0
1
2
3
4
5
Chromosome
1
2
3
4
5
6
7
8
9
10
11
12
13
14
15
16
17
18
19
20
21
22
24
26
28

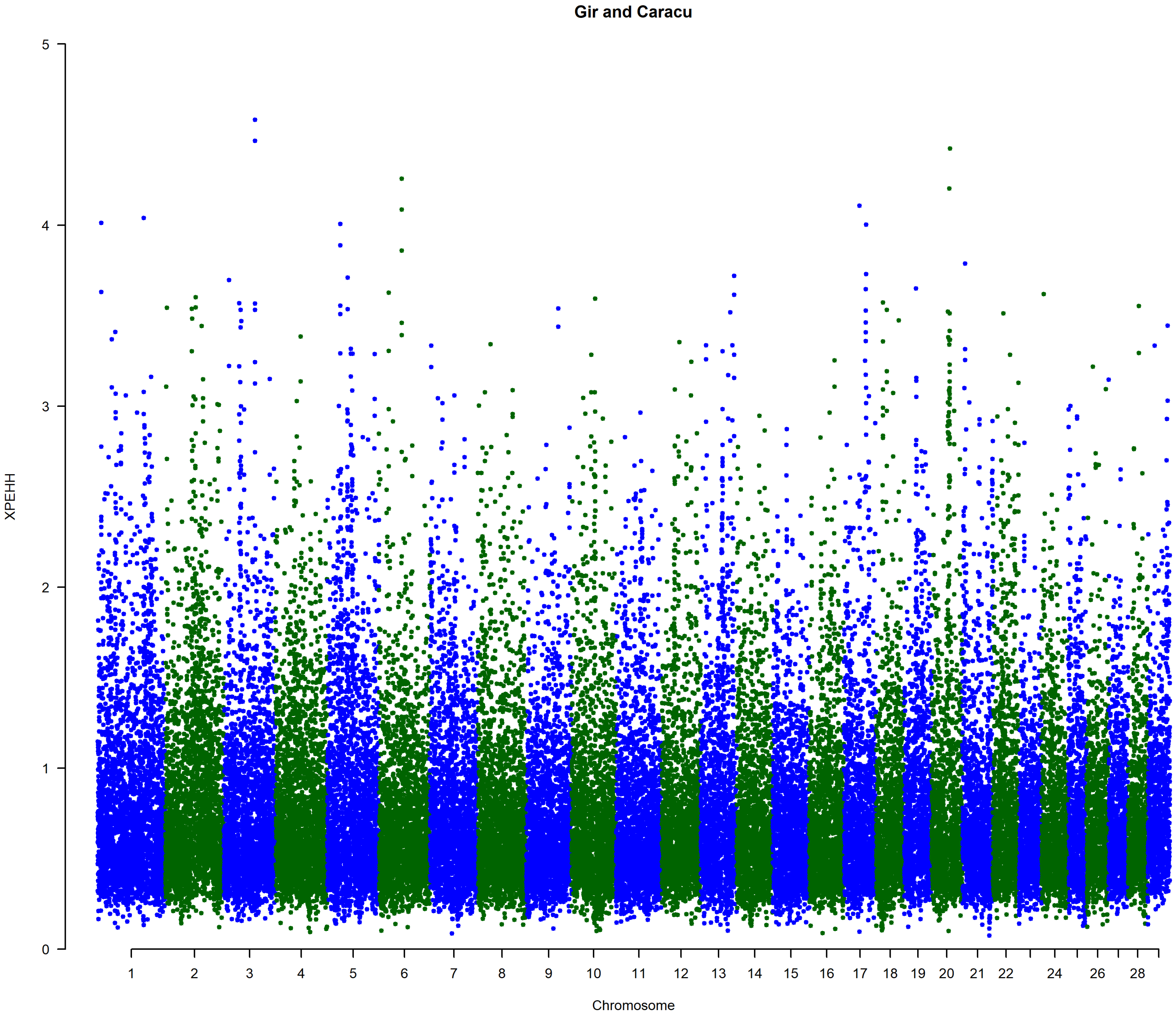

Gir and Caracu
XPEHH
0
1
2
3
4
5
Chromosome
1
2
3
4
5
6
7
8
9
10
11
12
13
14
15
16
17
18
19
20
21
22
24
26
28

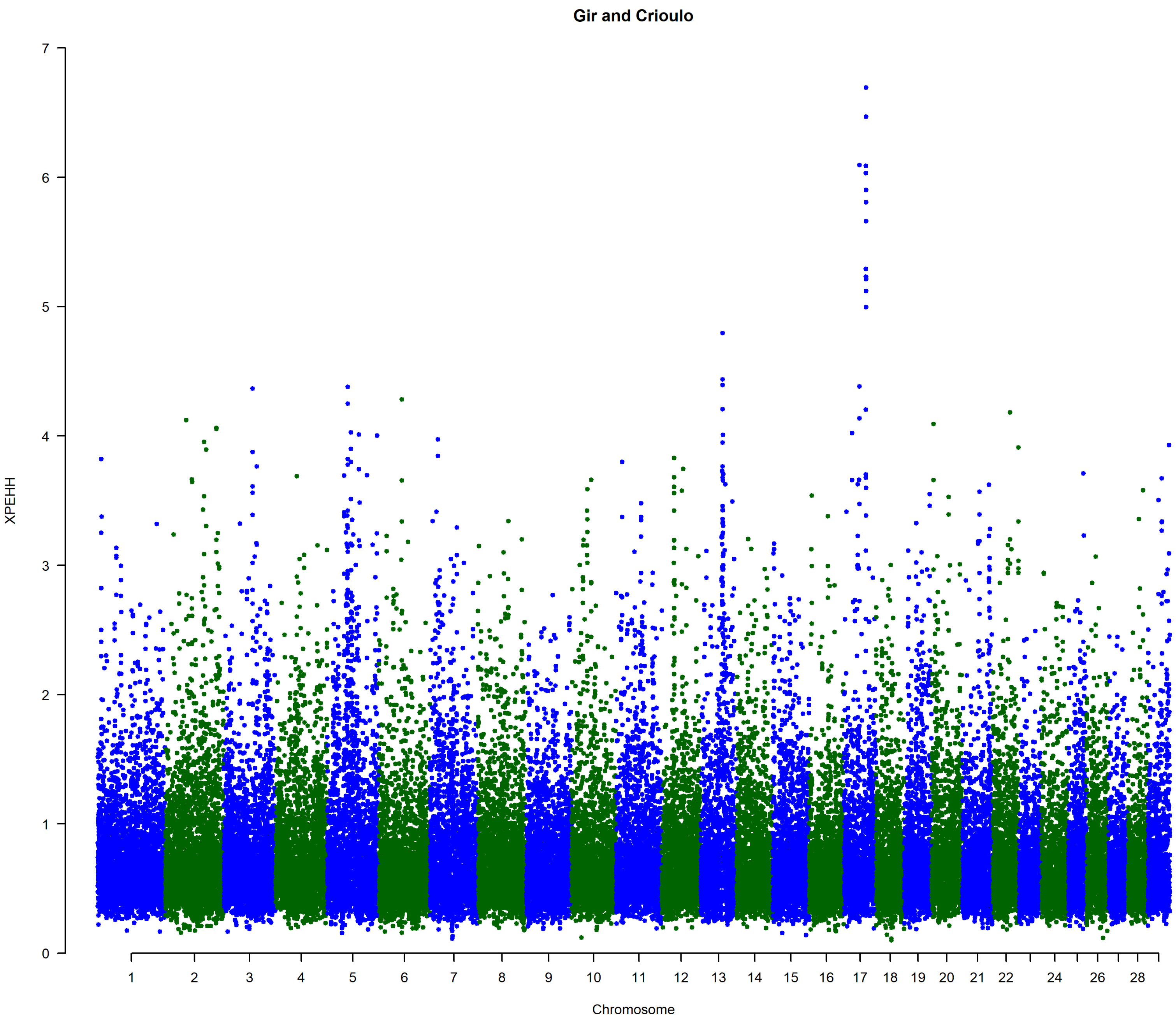

Gir and Crioulo
XPEHH
Chromosome
0
1
2
3
4
5
6
7
1
2
3
4
5
6
7
8
9
10
11
12
13
14
15
16
17
18
19
20
21
22
24
26
28

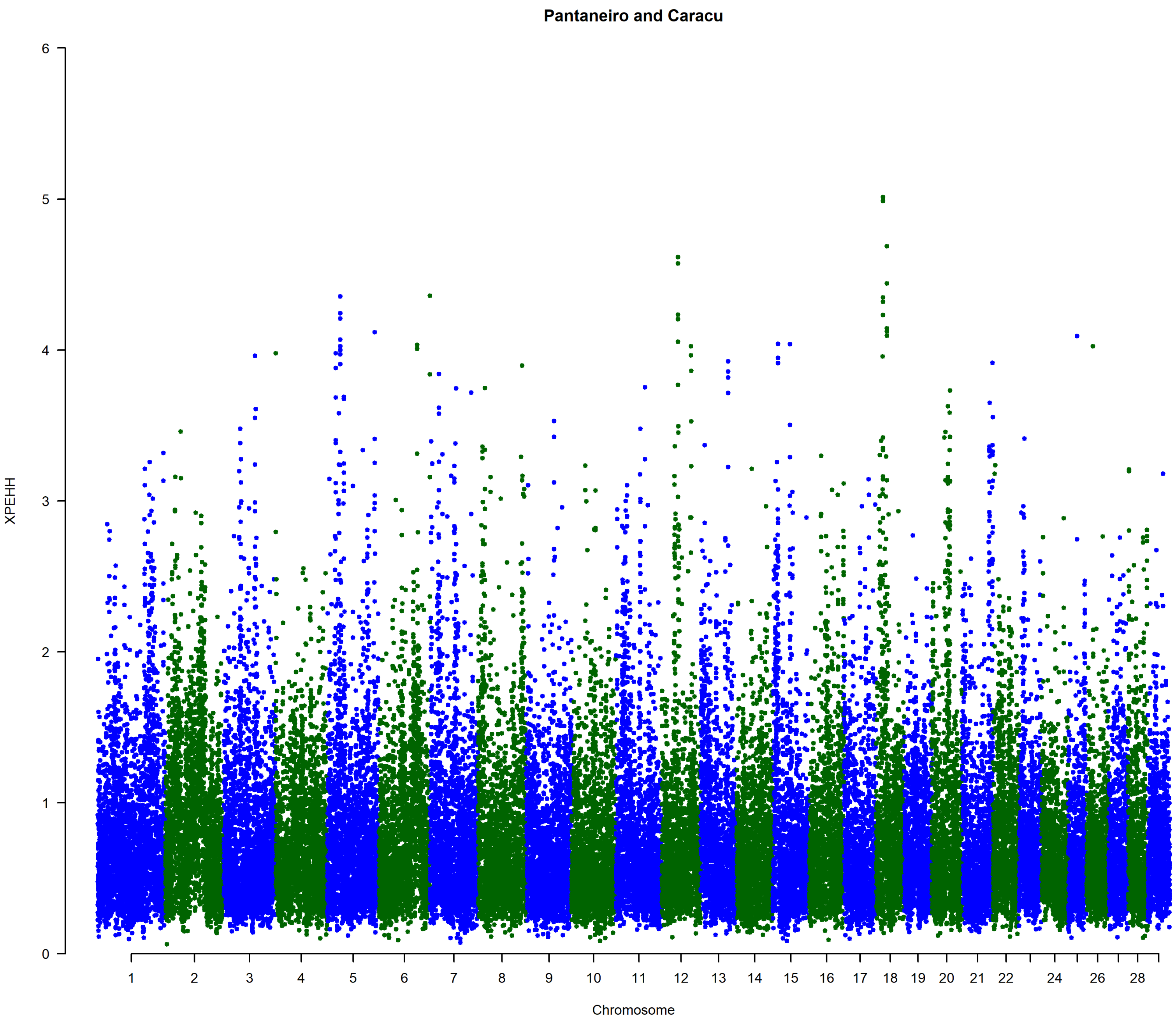
Pantaneiro and Caracu
XPEHH
Chromosome
0
1
2
3
4
5
6
1
2
3
4
5
6
7
8
9
10
11
12
13
14
15
16
17
18
19
20
21
22
24
26
28

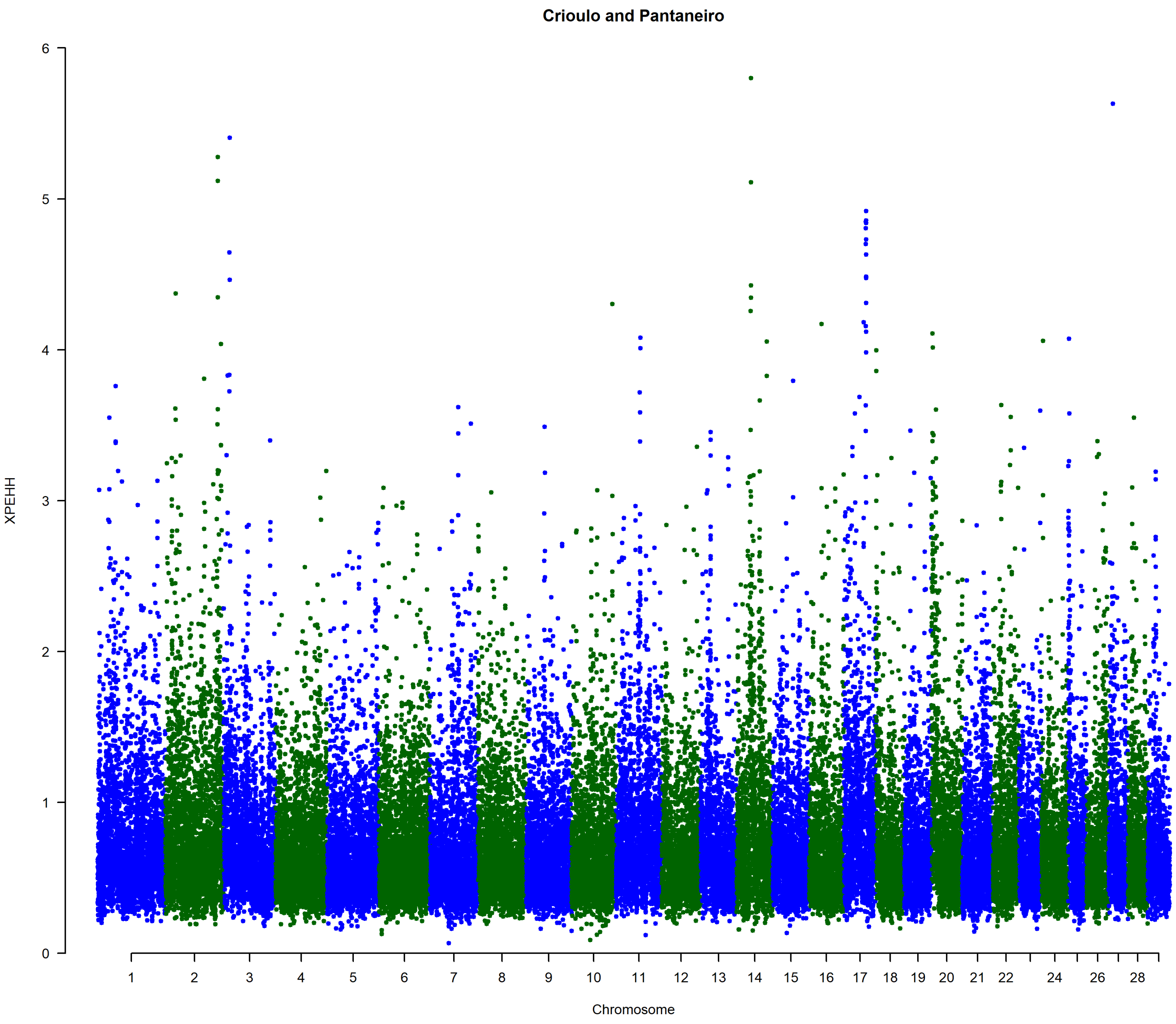
Crioulo and Pantaneiro
XPEHH
Chromosome
0
1
2
3
4
5
6
1
2
3
4
5
6
7
8
9
10
11
12
13
14
15
16
17
18
19
20
21
22
24
26
28

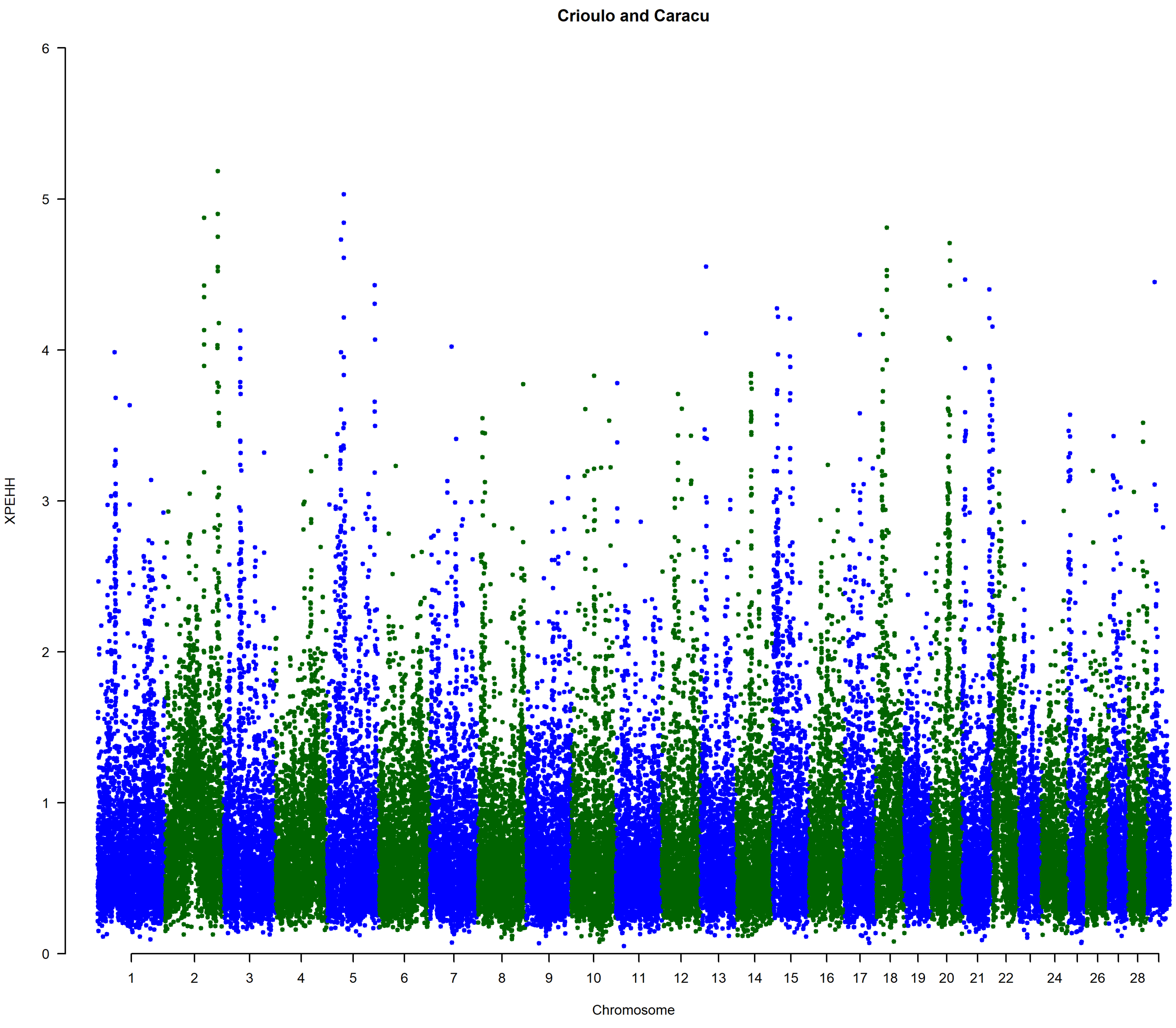
Crioulo and Caracu
XPEHH
0
1
2
3
4
5
6
Chromosome
1
2
3
4
5
6
7
8
9
10
11
12
13
14
15
16
17
18
19
20
21
22
24
26
28
